# Gene repositioning within the cell nucleus is not random and is determined by its genomic neighborhood

**DOI:** 10.1186/s13072-015-0025-5

**Published:** 2015-09-17

**Authors:** K. Laurence Jost, Bianca Bertulat, Alexander Rapp, Alessandro Brero, Tanja Hardt, Petra Domaing, Claudia Gösele, Herbert Schulz, Norbert Hübner, M. Cristina Cardoso

**Affiliations:** Department of Biology, Technische Universität Darmstadt, 64287 Darmstadt, Germany; Max Delbrück Center for Molecular Medicine, 13125 Berlin, Germany

**Keywords:** 3D-FISH measurements, Chromocenters, Genomic context, Gene position, Gene silencing, Heterochromatin proximity, MeCP2, Myogenesis, Nuclear periphery, Transcriptional profiling

## Abstract

**Background:**

Heterochromatin has been reported to be a major silencing compartment during development and differentiation. Prominent heterochromatin compartments are located at the nuclear periphery and inside the nucleus (e.g., pericentric heterochromatin). Whether the position of a gene in relation to some or all heterochromatin compartments matters remains a matter of debate, which we have addressed in this study. Answering this question demanded solving the technical challenges of 3D measurements and the large-scale morphological changes accompanying cellular differentiation.

**Results:**

Here, we investigated the proximity effects of the nuclear periphery and pericentric heterochromatin on gene expression and additionally considered the effect of neighboring genomic features on a gene’s nuclear position. Using a well-established myogenic in vitro differentiation system and a differentiation-independent heterochromatin remodeling system dependent on ectopic MeCP2 expression, we first identified genes with statistically significant expression changes by transcriptional profiling. We identified nuclear gene positions by 3D fluorescence in situ hybridization followed by 3D distance measurements toward constitutive and facultative heterochromatin domains. Single-cell-based normalization enabled us to acquire morphologically unbiased data and we finally correlated changes in gene positioning to changes in transcriptional profiles. We found no significant correlation of gene silencing and proximity to constitutive heterochromatin and a rather unexpected inverse correlation of gene activity and position relative to facultative heterochromatin at the nuclear periphery.

**Conclusion:**

In summary, our data question the hypothesis of heterochromatin as a general silencing compartment. Nonetheless, compared to a simulated random distribution, we found that genes are not randomly located within the nucleus. An analysis of neighboring genomic context revealed that gene location within the nucleus is rather dependent on CpG islands, GC content, gene density, and short and long interspersed nuclear elements, collectively known as RIDGE (regions of increased gene expression) properties. Although genes do not move away/to the heterochromatin upon up-/down-regulation, genomic regions with RIDGE properties are generally excluded from peripheral heterochromatin. Hence, we suggest that individual gene activity does not influence gene positioning, but rather chromosomal context matters for sub-nuclear location.

**Electronic supplementary material:**

The online version of this article (doi:10.1186/s13072-015-0025-5) contains supplementary material, which is available to authorized users.

## Background

Nuclear topology, in particular, the 3D landscape of the genome within the nucleus, has come into focus as a regulator of genome activity [1] with heterochromatin as a key player [2–4]. First evidence that heterochromatin might be a silencing compartment was provided by Mueller’s position effect variegation (PEV) experiments in 1930 [5], demonstrating that rearrangement of genes near the heterochromatin in *Drosophila* causes gene silencing. Position effect variegation affects genes on the same chromosome (*cis*) as well as genes on different chromosomes (*trans*) [6]. Moreover, the effects of heterochromatin on gene activity were suggested in, e.g., mouse [7–9], *Drosophila melanogaster* [10], *Caenorhabditis elegans* [11], *Saccharomyces cerevisiae* [12] *Schizosaccharomyces pombe* [13] and in *Plasmodium falciparum* [14], and seem to be an evolutionarily conserved feature [15, 16].

Heterochromatin can be found in essentially all eukaryotes, but its distribution and composition differ from species to species. In general, heterochromatin can be subdivided into two subgroups which differ in composition and location within the nucleus [17]. Facultative heterochromatin is cell-type specific, well documented by electron microscopy and found lining the lamina at the inside of the nucleus. Henceforth, we use the terms nuclear periphery and facultative heterochromatin interchangeably. Constitutive heterochromatin is found at and around centromeres (centric and pericentric heterochromatin) and able to form clusters of multiple chromosomes in some species. In mouse, pericentric heterochromatin clusters are located distant from the periphery inside the nucleus. These so-called chromocenters consist of highly condensed, repetitive DNA, are mostly transcriptionally silent and have been described in mouse [18, 19], *Drosophila* [10] and plants [20–22].

Both forms of heterochromatin (chromocentric and peripheral) have been hypothesized to act as silencing compartments. Experimental evidence for this hypothesis came from mouse lymphocyte maturation where Brown et al. [8] documented colocalization of inactive genes, but not active genes with chromocenters. Later studies performed in *Drosophila* additionally accounted for chromatin mobility by comparing the distance measurements of active and inactive gene loci to heterochromatin [10]. Several other reports provided further evidence of a positive correlation between gene silencing and either the distance to chromocenters [23] or the nuclear periphery [24, 25]. In addition, experiments in which ectopically tagged loci were artificially tethered to the nuclear lamina mostly resulted in the silencing of the respective locus [26, 27]. These studies, though, did not always observe a relocation of genes toward or away from chromocenters/nuclear periphery according to their expression status (reviewed in [28]). Several reasons might account for this fuzzy outcome. Firstly, different model systems and different genes were investigated. Second, the inherent challenge of 3D distance measurements was approached differently [17]. Hence, the variability of biological samples and the different technical approaches make the results difficult to compare, as common standards are not yet agreed upon [29]. Especially, morphological changes or differences need to be considered, as shape differences strongly influence the results of distance measurements. For example, spherical hematopoietic cells significantly differ from flat ellipsoid adherent cells. This shape difference increases the probability to be close to the periphery in flat cells compared to spheroid cells. Furthermore, the remodeling of heterochromatin seems to be a common feature of differentiation, and particular changes in chromocenter morphology are known to accompany the differentiation of mouse and human embryonic stem cells as well as mouse myoblasts [18, 19, 30–32]. The prevalence of chromatin reorganization during differentiation hints at a functional role of heterochromatin during this process. Nonetheless, studies that explicitly correct for nuclear morphology-associated changes when analyzing the influence of gene-to-heterochromatin distance on gene expression are still underrepresented. Another common bias of the studies so far is that, predominantly, the genes investigated were selected by candidate gene approaches. This candidate gene selection has served as a paradigm to elucidate different levels of gene regulation, but may, in fact, not reflect the way the whole genome is regulated.

Here, we reevaluated the impact of heterochromatin proximity on gene expression and additionally considered their genomic context. Using a well-established and characterized cellular differentiation system, we avoided the candidate gene analysis by performing a genome-wide transcriptional profile to identify up-/down-regulated and unchanged genes. As nuclei undergo significantly morphological changes during myogenic differentiation [18], we applied a single-cell-based normalization to all our 3D-FISH distance measurements [33]. Importantly, we also investigated the effect of induced heterochromatin reorganization in the absence of cellular differentiation. In a nutshell, we found that the gene’s neighborhood is far more influential in determining its nuclear positioning than the gene’s activity per se.

## Results and discussion

### Cellular systems for chromatin reorganization and respective gene selection based on transcriptional profiling

We tackled the controversial question of whether a gene’s location within the nuclear landscape and its proximity to heterochromatin influence its activity by comparing the location of differently expressed genes obtained from transcriptional profiling analysis. The latter provides an unbiased mode to select genes that are either up-regulated, down-regulated or not significantly changed in expression.

**Fig. 1:**
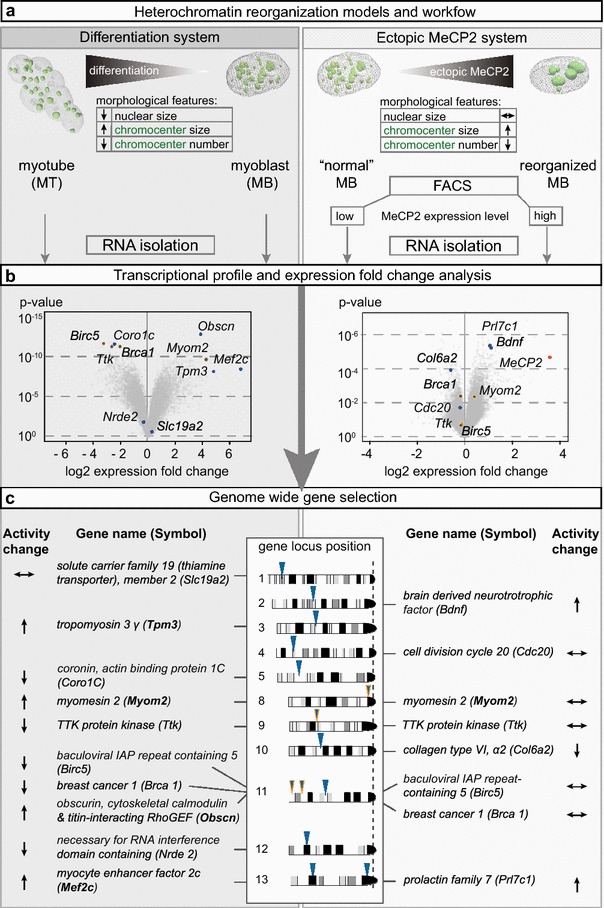
Genome-wide transcriptional profiling and gene selection. **a** Experimental design using two different cellular systems. On the *left*, a differentiation-based cell system and on the *right* a cell system based on transient ectopic MeCP2 expression. Both systems lead to a chromatin reorganization resulting in less and larger chromocenters. Both systems were used for gene expression profiling. **b** Results from the transcriptional profiling of the differentiation system (*left*) and the ectopic MeCP2 expression system (*right*) are presented in *volcano plots* (expression fold change versus statistical significance of the change). Genes selected for further analysis are depicted in *blue*. Selected genes shared in both conditions are outlined in *orange*. The expression change of the MeCP2 gene itself (11 fold) is depicted in *red*. As expected, the highest expression difference in low versus high MeCP2-expressing cells was MeCP2 itself. **c** The physical position of all selected genes on the mouse chromosomes with their full names. *Arrows* indicate if genes were up-, down- or unregulated during differentiation (*left*) or MeCP2 ectopic expression (*right*). *Bold gene names* indicate the myogenic genes according to the gene ontology classification

For that, we chose first the mouse myogenic in vitro differentiation system and compared gene expression profile of undifferentiated mouse myoblasts (MB) to differentiated myotubes (MT) (Fig. 1a, Additional file 1: Figure S1; differentiation system). This classic differentiation system is characterized by global changes in gene expression associated with distinct morphological alterations and well-described heterochromatin reorganization [18, 34, 35]. In particular, the syncytial morphology of the myotubes allows an unquestionable and direct identification of the differentiated state by contrast microscopy with no need whatsoever for additional molecular marking and immuno-FISH (Additional file 1: Figure S1). Accompanying differentiation, the average number of constitutive heterochromatin domains (called chromocenters) decreases in number and increases in size (Fig. 1a). Ectopic MeCP2 that is known to be necessary and sufficient for heterochromatin reorganization mimics this effect in a dose-dependent way in the absence of cellular differentiation [18, 36]. Therefore, to study the effects of heterochromatin reorganization decoupled from the general differentiation program, we next used the same myoblast cell line transfected with MeCP2-YFP and FACSorted (Fig. 1a; ectopic MeCP2 system). In both systems, low MeCP2 levels were accompanied by a high number of small chromocenters, while high MeCP2 levels were associated with a reduced number of larger chromocenters (Fig. 1a). Both systems provided us with the opportunity to investigate gene positioning dependent on chromatin reorganization with and without differentiation-associated large-scale gene expression changes.

We performed a genome-wide transcriptional analysis and profiled undifferentiated myoblasts, differentiated myotubes as well as low- and high-level MeCP2-expressing cells for their gene expression (GEO series accession number GSE69087). Subsequently, we analyzed the differentiation (MT versus MB) and the ectopic MeCP2 expression system (high versus low MeCP2 levels) for significant changes in gene expression and considered statistical (*p* values) and biological (gene expression fold changes) parameters (Fig. 1b). In parallel, we took advantage of “DAVID” (Database for Annotation, Visualization And Integrated Discovery; http://david.abcc.ncifcrf.gov/, [37, 38]) and could confirm the quality of our expression data. We also validated the quality of our in vitro differentiation by (1) morphological evaluation and (2) analyzing the expression data and finding myogenic-related genes up- and proliferation-related genes down-regulated (Additional file 1: Figure S1). The ectopic MeCP2 expression system showed lower global expression changes (with the obvious exception of the ectopically expressed MeCP2) as compared to the differentiation system (Fig. 1b). This observation agrees with previous expression data in MeCP2-deficient/mutated mouse and human brain [39, 40] and lymphocytes from patients [41, 42].

Based on the statistical significance (*p* value) of the observed expression changes (fold change), we further focused on 14 genes, distributed throughout the mouse genome: 10 genes within the differentiation system and 8 genes within the ectopic MeCP2 expression system, including 4 genes shared by both systems (Fig. 1c). Selected genes either showed highly significant up- (indicated by an upward arrow) or down-regulation (indicated by a downward arrow) or insignificant statistical changes in those chosen as the control group (indicated by an horizontal arrow). The ten genes of the differentiation system included myogenic-specific genes (*Mef2c*, *Myom2*, *Obscn, Tpm3*) and genes unrelated to myogenesis (*Birc5*, *Brca1, Coro1c*, *Nrde2, Slc19a2*, *Ttk*) according to the gene ontology classification. In addition to *Birc5*, *Brca1,Myom2* and *Ttk* shared by both systems (Fig. 1c; names in bold font and chromosomal location highlighted), *Bdnf*, *Cdc20*, *Col6a2* and *Prl7c1* were analyzed in the ectopic MeCP2 expression system and considered to be genes unrelated to the differentiation program. Figure 1c summarizes: (1) selected genes’ full name and abbreviation for each system as well as genes selected in both systems; (2) their chromosomal location; (3) their change in gene expression upon differentiation and ectopic MeCP2 expression.

**Fig. 2:**
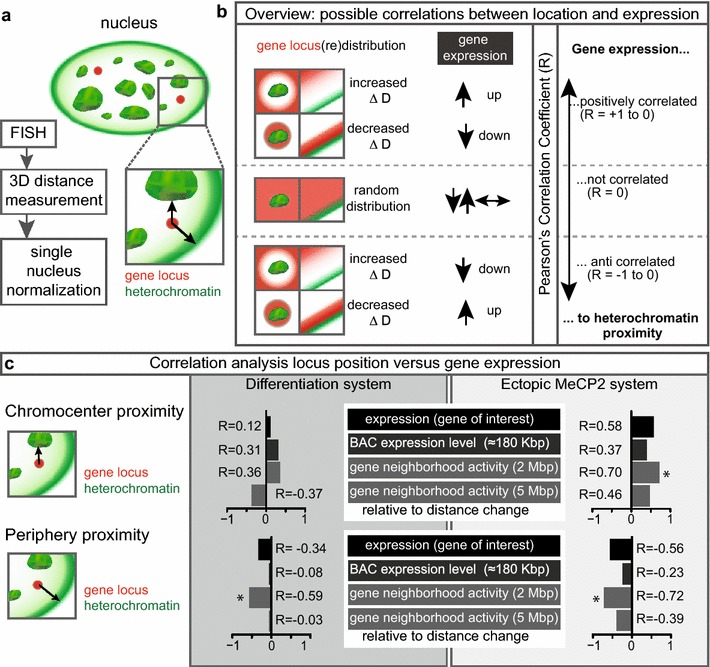
Gene repositioning relative to heterochromatin compartments. **a** Graphical summary of the experimental procedure with gene locus detected by 3D FISH depicted as *red dots* and heterochromatin compartments in *green*. The shortest 3D distances to the constitutive (chromocenters) and peripheral heterochromatin (*black arrows*) were measured and single cell normalized as described in the “Methods”. **b** Rationale and visual explanation of possible Pearson’s correlation coefficients (*R*) relating gene expression changes (up-regulated, down-regulated or no expression change) to changes (Δ) in gene locus proximity to heterochromatin (chromocenters at the *left* and periphery at the *right*). A positive correlation (*R* = 0 to +1) indicates movement to heterochromatin upon down-regulation or vice versa, confirming heterochromatin as a silencing compartment. A negative correlation means that genes move closer to heterochromatin upon up-regulation (or away upon down-regulation). A negative correlation (*R* = 0 to −1) does not support the hypothesis of heterochromatin as a silencing compartment. **c** Results of the correlation analysis of locus repositioning (relative to chromocenters and to the periphery, as indicated) versus changes in gene expression upon differentiation and ectopic MeCP2 expression. Expression changes (during myogenesis and upon ectopic MeCP2 expression) are correlated for mean normalized distances at different scales: gene locus of interest, whole BAC, 2- and 5-Mbp genomic domains centered around the gene of interest. *Significant correlation (*p* < 0.05) (Table 1)

For each system and condition (i.e., MB or MT, low or high MeCP2) 3D FISH experiments were performed and at least 47 nuclei were analyzed. Using our previously developed 3D distance measurement tool [33], we measured the gene loci–heterochromatin distance (Fig. 2a; Additional file 1: Tables S1–S4). To further analyze and compare 3D distances corrected for morphological differences between conditions, we applied a single nucleus-based normalization algorithm described before [33]. In brief, by simulating 10,000 random points followed by 3D distance measurements toward (1) the nearest chromocenter surface (defined as DAPI dense signals) and (2) the nuclear periphery (defined as edge of the DAPI signal), we generated a background distribution for each analyzed nucleus. In a subsequent step, we normalized the actual gene locus–heterochromatin distances to the same individual cell background distribution generated in the step before. Finally, we correlated gene expression data and normalized 3D distances using a Pearson’s correlation coefficient (R) (Fig. 2a, b).

### Gene repositioning to heterochromatin and gene expression

We next tested if there is a correlation between gene expression and gene (re)positioning. As the nuclei undergo large-scale morphological changes during differentiation (Fig. 1a, Additional file 1: Figure S2), it is mandatory to consider those changes and their effect on any gene–heterochromatin distances [33]. Therefore, we first normalized the distances for morphological differences to compensate for nuclear changes in shape and size (Additional file 1: Tables S1–S4; Figures S3–S6).

To evaluate the correlation between normalized 3D distance changes (Additional file 1: Tables S1–S4) and gene expression changes (Additional file 1: Figure S7), we calculated the Pearson’s correlation coefficient that varies between *R* = 1 (positive correlation) and *R* = −1 (anti-correlation). A large variation within the data set results in a Pearson’s correlation coefficient of *R* = 0 or values close to 0 (no correlation). Hence, if up-regulated genes increased their gene–heterochromatin distance and down-regulated genes move closer to the heterochromatin, movement and gene expression levels would be correlated and yield values close to *R* = 1 (Fig. 2b). If, on the other hand, up-regulated genes decreased their gene–heterochromatin distance and down-regulated genes move away from the heterochromatin, movement and gene expression levels would be anti-correlated and yield values close to *R* = −1 (Fig. 2b). If a gene locus did not significantly change its location upon a change in expression or vice versa, this would result in *R* = 0 (Fig. 2b).

In the differentiation system and ectopic MeCP2 expression systems, for relations between gene expression change and change of gene–chromocenter distance, we obtained weak to moderate positive correlation values of *R* = 0.12 (*p* = 0.37) and *R* = 0.58 (*p* = 0.07), respectively (Fig. 2c; Table 1). Although these correlations may have biological relevance, they are statistically nonsignificant. The fact that genes in the neighborhood may have a different expression level than the locus selected (see Additional file 1: Figures S7, S8) may constrain the movement of the locus itself. Therefore, we further considered the gene activity within the genomic neighborhood. We calculated the average gene activity within the whole BAC used as a probe as well as in 2- and 5-Mbp neighborhoods centered around the target gene (1 and 2.5 Mbp up- and downstream; see Additional file 1: Tables S5, S6). Even if considering the average gene expression change of the whole neighborhood at the different scales, we observed no significant correlation between gene activity and gene–chromocenter distance except for measurements considering the 2-Mbp genomic region (Fig. 2c). The latter yielded significant (*p* = 0.03) correlation (*R* = 0.7) within the ectopic MeCP2 expression system (Fig. 2c; Table 1). Indeed, at all scales (gene of interest to 5 Mbp), there was a general tendency—though mostly not statistically significant—to positive correlation between gene expression change and proximity to chromocenters. Therefore, we conclude that gene activity is mostly not related to proximity or positional changes toward constitutive heterochromatin. Studies of gene silencing and localization to chromocenters have yielded inhomogenous outcomes. Some studies indicated gene silencing correlated with chromocenter proximity (e.g., [8]), whereas others showed either no correlation or negative correlation (e.g., [43]). Most differences have been attributed to either the cell type or species, or the particular gene loci studied. Our data would favor a scenario compatible with gene silencing being not determined by proximity to constitutive heterochromatin. Nonetheless, weak to moderate non-statistically significant correlation could still have biological consequences.

**Table 1:** Pearson’s correlation analysis of locus position and gene expression

Condition		Lower CI	Upper CI	*R*	*p* value
(A) Gene of interest					
Differentiation	Locus–periphery	−1	0.2587	−0.3425	0.1663
	Locus–chromocenter	−0.4619	1	0.121	0.3692
Ectopic	Locus–periphery	−1	0.1083	−0.556	0.07627
	Locus–chromocenter	−0.0698	1	0.582	0.06501
(B) BAC (≈180 kbp)					
Differentiation	Locus–periphery	*−1*	0.4907	−0.085	0.4082
	Locus–chromocenter	−0.2949	*1*	0.307	0.1937
Ectopic	Locus–periphery	−1	0.3716	−0.227	0.2638
	Locus–chromocenter	−0.2237	1	0.375	0.1429
(C) 2-Mbp region					
Differentiation	Locus–periphery	*−1*	−0.0516	−0.587	0.0372
	Locus–chromocenter	−0.2413	*1*	0.359	0.1543
Ectopic	Locus–periphery	−1	−0.1764	−0.723	0.0214
	Locus–chromocenter	0.1386	1	0.704	0.0257
(D) 5-Mbp region					
Differentiation	Locus–periphery	*−1*	0.2243	−0.374	0.1433
	Locus–chromocenter	*−1*	0.5311	−0.030	0.4672
Ectopic	Locus–periphery	−1	0.317	−0.386	0.1724
	Locus–chromocenter	−0.0235	1	0.459	0.1261

Differentiation and ectopic MeCP2 systems were analyzed relating normalized locus positions and maximum gene expression values (see Fig. 2). Different region sizes were analyzed: (A) single gene level; (B) BAC level, corresponding to an average of 180 kbp; (C) genomic 2-Mbp window; (D) genomic 5-Mbp window. Pearson’s coefficients (*R*) are given together with upper and lower confidence intervals (CI) and *p* values for each condition as indicated

Next, we analyzed a putative relation of gene activity and proximity to heterochromatin at the nuclear periphery. In contrast to the tendency to have positive correlation in the previous setting, we found only anti-correlation. Using the normalized distances and expression changes, we calculated a correlation coefficient of *R* = −0.34 (*p* = 0.17) and *R* = −0.56 (*p* = 0.08) for differentiation and ectopic MeCP2 expression system, respectively (Fig. 2c; Table 1). This negative, albeit non-statistically significant, correlation indicates that up-regulated genes are repositioned closer to the periphery, whereas down-regulated genes are farther away from the periphery. To exclude neighborhood effects, we correlated the surrounding gene activity as above with repositioning and obtained again negative correlations (Fig. 2c). We found only significant anti-correlation (*R* = −0.59, *p* = 0.04 and *R* = −0.72, *p* = 0.02 for differentiation and ectopic MeCP2 expression system, respectively) within the 2-Mbp genomic region (Fig. 2c; Table 1). Therefore, we conclude that gene activity is unexpectedly associated with proximity or positional changes toward peripheral heterochromatin. This outcome differs from previous reports, e.g., analyzing immunoglobulin genes during development of mouse lymphocytes [44], but agrees with other reports describing the opposite (e.g., [45]). In fact, the same gene in human and mouse cells have been shown to differ concerning nuclear localization and expression state [46, 47]. Our data support the concept that gene activity is correlated with proximity to the nuclear periphery and does not agree with the more established concept of nuclear periphery as a silencing compartment.

In view of these results, mouse heterochromatin may not be considered as a general silencing compartment for single genes or their genomic neighborhoods. While gene–chromocenters distance did correlate with gene regulation, nuclear periphery proximity was anti-correlated (Fig. 2c). Interestingly, Blobel [48] suggested already in 1985 a spatial correlation of active genes and nuclear pores. This theory was dubbed the “gene-gating hypothesis” and stated that active genes would be close to nuclear pores to facilitate efficient transport of their mRNA out of the nucleus. Recent results in yeast point to the same mechanism (also reviewed in [49–51]). Since our data do not allow for discrimination between lamina and nuclear pore association, this might explain our observation in that the up-regulated genes could move toward the nuclear pores. However, we cannot exclude that other additional factors might be able to overrule the simple correlation between gene expression and heterochromatin distance and influence gene positioning within the nucleus.

### Gene position within the nucleus is not random and is determined by RIDGE properties

To test if our results were not reflecting mere random gene positioning within the nucleus in general, we calculated a random distribution. Random points were uniformly simulated throughout the 3D nucleus and distance measurements were performed as previously described. The acquired simulated data wére collected. Normalized distances were binned in 0.25 steps and their relative frequency was calculated. Next, to test for divergence from a random distribution (i.e., relative frequency of 25 % for each bin) the Chi-square value was calculated (Additional file 1: Table S7). From all experimental measurements, only 8 % showed a random distribution (Additional file 1: Table S7, gray shading). These results emphasize that genes are not randomly positioned within the nucleus, but according to specific properties.

To determine whether and which other factors might influence gene positioning and potentially overrule positional changes due to gene expression, we investigated the role of different genomic features. We considered the following genomic properties within a 2- and 5-Mbp neighborhood surrounding the gene: (1) gene density (number of genes), (2) number of CpG islands, (3) % GC content (fraction of GC within the sequence), (4) density of short interspersed nuclear elements (SINE) (percentage of covered sequence) and (5) density of long interspersed nuclear elements (LINE) (percentage of covered sequence). The genomic properties were summarized for the differentiation system (Additional file 1: Table S5) and for ectopic MeCP2-expressing cells (Additional file 1: Table S6), for a core neighborhood spanning 2 Mbp and for an extended 5-Mbp neighborhood. As we obtained similar results for both, 2- and 5-Mbp ranges, we concentrated on the 2-Mbp window neighborhood for further evaluation. Furthermore, in the previous analysis, only the 2-Mbp region gave statistically significant outcomes (Fig. 2c; Table 1).

Concerning the selected genomic features, CpG islands were defined as regions with a minimal length of 500 bp, a GC content of 50 % or above and an observed CpG/expected CpG ratio of 0.60 or higher [52]. CpG islands are associated with 70 % of all gene promoters in vertebrate genomes [53]. Hence, high numbers of CpG islands could serve as indicators for active gene transcription and their occurrence might correlate with greater distances to potentially repressive compartments such as chromocenters and the nuclear periphery. The additional monitored retroposons, including LINEs and SINEs, are distributed throughout the mouse genome (37 %) [54] and originally considered to be “junk DNA”. However, already in the 1960s, it has been suggested that noncoding RNAs might be regulators of gene transcription [55, 56] and more recent studies provided evidence for a functional role of noncoding RNA transcribed from heterochromatin [57].

**Fig. 3:**
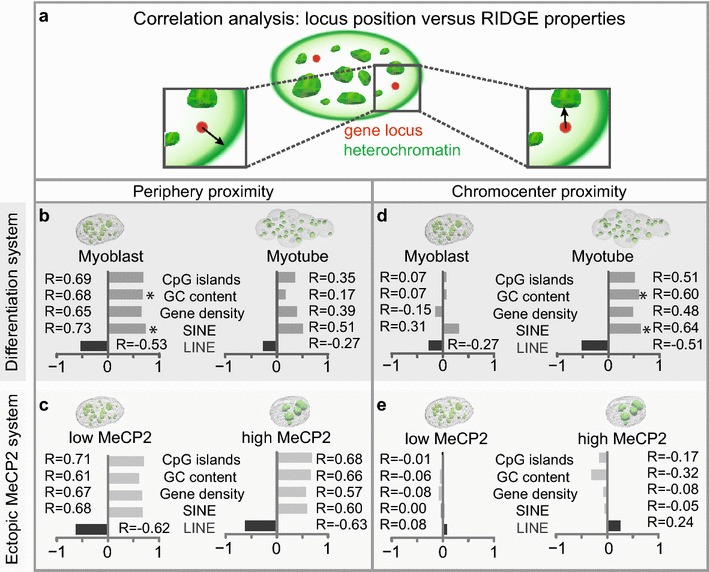
RIDGE properties determine gene position. **a** Schematic representation of the gene locus distance measurements to chromocenters (*right*) and nuclear periphery (*left*). **b**–**e** Results of the correlation analysis of locus position versus RIDGE (*light gray bars*) as well as anti-RIDGE (*dark gray bars*) properties upon differentiation and ectopic MeCP2 expression, as indicated. *Significant correlation (*p* < 0.05) (Table 2)

The combination of the above-mentioned genomic features serves as a marker for regions of increased gene expression (RIDGEs) [58]. RIDGEs contain housekeeping genes which are broadly expressed in all tissues [59] and on the linear genome. RIDGEs alternate with anti-RIDGES. They are defined as regions with high gene density, high GC content, high percentage of CpG islands, high numbers of SINEs and low numbers of LINEs, while anti-RIDGES are defined by the exact opposite. Therefore, we could use these selected genomic properties as marker for RIDGEs in a defined neighborhood to elucidate positioning of these regions relative to heterochromatin during differentiation and ectopic MeCP2 expression (Fig. 3).

In contrast to gene transcription, these are genomic features and, hence, do not change during differentiation. Therefore, gene locus position rather than repositioning was considered in each biological condition (Additional file 1: Tables S1–S4). Next, we correlated gene–periphery as well as gene–chromocenter distances with each of these genomic properties (Fig. 3b–e; Table 2). Positive correlation was defined as genomic regions with high RIDGE properties correlating with larger distances to heterochromatin (chromocenters and nuclear periphery) and vice versa.

**Table 2:** Pearson’s correlation analysis of RIDGE properties versus normalized mean distances

				Lower CI	Upper CI	*R*	*p* value
(A) Differentiation system							
Locus–periphery	Myoblast (MB)	RIDGE	Number of CpG islands	−1	0.900	0.692	0.987
			% GC content	0.204	1	0.680	0.015
			Genes within region	−1	0.884	0.648	0.979
			% SINE	0.307	1	0.735	0.008
			*% LINE*	*−1*	*0.030*	*−0.531*	*0.057*
	Myotube (MT)	RIDGE	Number of CpG islands	−0.248	1	0.352	0.159
			% GC content	−0.425	1	0.167	0.322
			Genes within region	−0.206	1	0.391	0.132
			% SINE	−0.060	1	0.509	0.066
			*% LINE*	*−1*	*1*	*−0.271*	*0.224*
Locus–chromocenter	Myoblast (MB)	RIDGE	Number of CpG islands	−0.502	1	0.070	0.424
			% GC content	−0.501	1	0.071	0.423
			Genes within region	−0.645	1	−0.145	0.655
			% SINE	−0.290	1	0.312	0.190
			*% LINE*	*−1*	*0.329*	*−0.273*	*0.223*
	Myotube (MT)	RIDGE	Number of CpG islands	−0.059	1	0.510	0.066
			% GC content	0.071	1	0.600	0.033
			Genes within region	−0.105	1	0.475	0.083
			% SINE	0.128	1	0.635	0.024
			*% LINE*	*−1*	*0.054*	*−0.514*	*0.064*
(B) Ectopic system							
Locus–periphery	Low MeCP2 level	RIDGE	Number of CpG islands	−1	0.925	0.709	0.975
			% GC content	−1	0.894	0.609	0.946
			Genes within region	−1	0.913	0.668	0.965
			% SINE	−1	0.916	0.680	0.965
			*% LINE*	*−0.899*	*1*	*−0.624*	*0.951*
	High MeCP2 level	RIDGE	Number of CpG islands	−1	0.917	0.683	0.969
			% GC content	−1	0.911	0.663	0.964
			Genes within region	−1	0.883	0.575	0.932
			% SINE	−1	0.891	0.597	0.941
			*% LINE*	*−0.899*	*1*	*−0.626*	*0.952*
Locus–chromocenter	Low MeCP2 level	RIDGE	Number of CpG islands	−0.634	1	−0.012	0.512
			% GC content	−0.662	1	−0.060	0.556
			Genes within region	−0.670	1	−0.075	0.570
			% SINE	−0.628	1	−0.002	0.502
			*% LINE*	*−1*	*0.672*	*0.079*	*0.573*
	High MeCP2 level	RIDGE	Number of CpG islands	−0.717	1	−0.165	0.652
			% GC content	−0.788	1	−0.318	0.779
			Genes within region	−0.673	1	−0.081	0.576
			% SINE	−0.658	1	−0.053	0.550
			*% LINE*	*−1*	*0.755*	*0.244*	*0.720*

Overview of Pearson’s correlation analysis in the differentiation (A) and ectopic MeCP2 system (B) (Fig. 3). For a 2-Mbp window around the gene of interest, the normalized mean distances toward the indicated heterochromatin compartment were correlated to RIDGE and anti-RIDGE properties. RIDGE properties include: number of CpG island, % of GC content, number of genes within the region and % of SINEs; % of LINES (italics) are defined as anti-RIDGES. For each correlation *p* values, upper and lower confidences were calculated

The outcome of the analysis of locus distances to the periphery are given in Fig. 3 and all numerical values are listed in Table 2. In myoblast and myotubes, we observed a positive correlation of the gene’s location with the number of CpG islands, GC content, gene density and SINEs (i.e., RIDGE properties), whereas we observed an anti-correlation with LINEs (i.e., anti-RIDGE property; Fig. 3b; Table 2). To determine whether RIDGE exclusion from the nuclear periphery is an artifact of chromatin reorganization, we compared these results to the ones in cells transiently expressing MeCP2, which mimics only the architectural chromatin remodeling during differentiation. The latter revealed the same pattern of correlations (Fig. 3c; Table 2), emphasizing that RIDGE exclusion from the nuclear periphery is a general feature and not due to cellular differentiation.

Performing the same correlation analysis for chromocenter proximities resulted in a very different outcome (Fig. 3d, e; Table 2). Distances to chromocenters in myoblasts showed no correlation, while we were able to observe an exclusion of RIDGEs from chromocenters in myotubes (Fig. 3d). This difference in myoblasts and myotubes hints at a differentiation-specific role. To exclude that heterochromatin remodeling plays a role, we used the MeCP2-expressing cells as a control. Interestingly, high and low MeCP2-expressing cells exhibited the same general lack of correlation with genomic properties as observed in myoblasts (Fig. 3e). However, only the differentiation system, for some conditions and genomic features, showed statistically significant positive correlations (Fig. 3b, d; Table 2).

We conclude that RIDGEs are excluded from peripheral heterochromatin in general and from chromocenters in differentiated myotubes. It would be interesting to address in the future whether RIDGE exclusion from heterochromatin during differentiation could be a mechanism to safeguard the activity of differentiation-specific genes. Our outcome is in agreement with hybridization results using LINE and SINE elements in mouse tissue and mouse fibroblasts [15], which clearly show SINE sequences within the interior lined by LINE elements at the periphery of the nucleus.

## Conclusion

Taken together, our genome-wide analyses underline that genes are positioned in a nonrandom pattern throughout the nucleus. We could establish that the proximity of genes to heterochromatin cannot generally be equated with gene silencing. In fact, gene activity rather than silencing is associated with proximity toward peripheral heterochromatin. However, we found a general exclusion of genomic regions with RIDGE properties from peripheral heterochromatin. Remarkably, this exclusion is differentiation independent with regard to the nuclear periphery, but not so relative to constitutive heterochromatin. One should consider that the name RIDGE, albeit implying potential for gene expression, is based on immutable hardwire DNA features, which do not imply gene expression in a particular cellular system. The latter is dependent on the cellular system and influenced by a variety of factors, e.g., differentiation, cell cycle, metabolic state, aging and stress. In summary, the nonrandom position of genes in the nucleus is based on their genomic context, which overrules the influence of the individual gene expression. Future studies should aim to elucidate the evolutionary conservation of gene positioning, its dependence on the genomic context and its pathophysiological relevance.

## Methods

### Cell culture and differentiation

Pmi 28 mouse myoblasts [34, 35] were cultured and differentiated into myotubes as described previously [18]. Briefly, for differentiation, 8 × 10^5^ myoblast cells were seeded on 100 mm Ø dishes and cultured for 4–7 days until the formation of large polynucleated myotubes became visible (details are provided in the Additional file 1: Figure S1). For subsequent 3D fluorescence in situ hybridization (FISH) experiments, cells were plated onto sterile glass cover slides and treated as described below.

### Transfection, FACSorting, RNA preparation and cDNA synthesis

Pmi 28 myoblasts were transfected with a mammalian expression construct coding for YFP-tagged rat MeCP2 [18] either using Transfectin reagent (Bio Rad, München, Germany) or by nucleofection using the Amaxa Kit V solution and program B32 (Lonza, Köln, Germany), both according to the manufacturers’ advice. After standard cultivation overnight, transfected cells were washed twice in PBS-EDTA and detached by standard trypsin treatment. Subsequently, the resulting cell suspension was gently pelleted at 200 × g for 3 min and pellets were resuspended in sterile PBS for FACSorting.

Cells were sorted using the FACS Aria I (Becton–Dickinson, Franklin Lakes, NJ, USA) by gating the fluorescent intensity into high (fluorescent intensity mean 322, hereafter termed R4) and low (fluorescent intensity mean 247, hereafter termed R5) MeCP2-expressing fractions, making up 8 or 25 % of all cells, respectively.

RNA was then prepared from all four conditions (myoblasts/myotubes, low/high MeCP2-expressing cells) and used for cDNA synthesis. For total RNA preparation, pellets with 6.5 × 10^5^ to 1.7 × 10^6^ cells were treated with TRIzol reagent (Invitrogen, Paisley PA4 9RF, UK) and the RNAeasy Mini kit (Qiagen, Valencia, CA 91355, USA) according to the manufacturers’ advice.

Depending on the total RNA yield, double-stranded cDNA was either synthesized using the One-Cycle cDNA Synthesis kit (Roche, Mannheim, Germany; yield 1–20 µg/µl) or the Two-Cycle kit (Invitrogen, Paisley PA4 9RF, UK; yield 10–100 ng/µl) following the manufacturers’ advice.

### Microarray analysis

The resulting cDNA was hybridized to the Affymetrix mouse 430 2.0 microarray, carrying 45,101 3′ probe sets per array. The data have been deposited in NCBI’s Gene Expression Omnibus and are accessible through GEO series accession number GSE69087 (http://www.ncbi.nlm.nih.gov/geo/query/acc.cgi?acc=GSE69087). For each sample set (undifferentiated MB, differentiated MT, high [R4] and low [R5] MeCP2-expressing cells), five independent experiments were performed. The quality of the hybridization and overall microarray performance was determined by visual inspection of the raw scanned data to exclude artifacts, scratches and bubbles. Further quality controls were performed using the GeneChip^®^ Operating Software report file (details given in the Additional file 1: Table S8). In particular, the statistics of the following parameters were checked: 3′/5′ signal ratio of GAPDH and β-actin, assay background and noise, and proportion and average expression value of detected genes. For each set, arrays were normalized individually, using a log-scale robust multi-array analysis (RMA), providing a consistent estimate of fold changes [60]. Additionally, a Nalimov test was performed to exclude outliers from further analysis (threshold: p = 0.001). Mean and standard deviation of the antilog RMA values were calculated and subsequently fold changes obtained. Next, an ANOVA test was performed over all sample sets as well as unpaired Student’s *t* tests over pairs of sets. Only genes exhibiting fold changes of high statistical significance (*p* ≤ 4 × 10^−6^) were chosen for further analysis.

### Bacterial artificial chromosomes and their gene expression analysis

Bacterial artificial chromosomes (BACs) were obtained from BAC-PAC resource center (Oakland, CA, USA, http://bacpac.chori.org) and used to generate biotin-dUTP-labeled DNA probes for 3D FISH.

**Table:** 

Gene name	BAC number
Baculoviral IAP repeat-containing 5	RP23-220P14
Breast cancer 1	RP23-222H10
Ttk protein kinase	RP24-211B11
Nrde2	RP24-117A2
Obscurin	RP23-113H6
Myocyte enhancer factor 2C	RP23-205E14
Tropomyosin 3, gamma	RP23-163L22
Procollagen, type VI, alpha 2	RP23-27P21
Prl7c1	RP23-155I17
Coronin, actin-binding protein 1C	RP24-156M14
Brain-derived neurotrophic factor	RP24-310A6
Myomesin 2	RP24-244I21
Solute carrier family 19 (thiamine transporter), member 2	RP24-158B1
Cdc20 like	RP23-118J14

Affymetrix gene expression analysis and translation to genomic coordinates were done on the basis of the Affymetrix 430.2 mouse annotation set (NetAffx version 35 based on the mouse reference genome assembly mm10). Annotated transcripts overlapping with the selected BACs according to their genomic coordinates (obtained from NCBI Map Viewer version 38; http://www.ncbi.nlm.nih.gov/projects/mapview/) were extracted from the obtained Affymetrix data. If multiple Affymetrix probe sets were linked to the same transcript, the maximally regulated transcript was chosen. Also, the percentage of overlap for each transcript with the BAC probe was calculated based on the genomic coordinates of NetAffx version 35 and the percentage of the BAC length that is covered by the corresponding transcript.

### DNA probes and (immuno) fluorescence in situ hybridization

Biotin-dUTP (Amersham, Buckinghamshire, UK)-labeled DNA probes were generated by nick translation using 2 µg BAC DNA and purified by sodium acetate/alcohol precipitation following standard protocols. Probes were finally resuspended to an approximate end concentration of 50 ng/µl in hybridization solution, containing 50 % formamide, 2xSSC (saline sodium citrate) buffer (pH 7.0) and 10 % dextran sulfate. In parallel to probe preparation, cells used for FISH experiments were fixed in 4 % paraformaldehyde in PBS (EM grade, Electron Microscopy Science, USA) for 10 min at 4 °C. Following a brief washing step in PBS, samples were permeabilized using 0.5 % Triton X-100/PBS for 20 min, treated for 10 min with 0.1 M HCl and incubated again in 0.5 % Triton X-100/PBS at room temperature for 20 min.

In case of MeCP2-YFP-expressing cells, the conditions for FISH eradicated the YFP signal and, thus, we performed immuno-FISH with antibodies to the MeCP2 protein [61]. For immuno-FISH experiments, cells were fixed as described and permeabilized in 0.25 % TritonX-100/PBS at 4 °C for 10 min. After incubation in blocking solution containing 4 % BSA (bovine serum albumin; Sigma-Aldrich, Germany) in PBS for 30 min, MeCP2 was detected with anti-MeCP2 antibodies as described previously [61] and visualized with suitable Alexa 488 secondary antibodies (Life Technologies, Germany). Before continuing with the FISH procedure, samples were post-fixed using 1 % paraformaldehyde/PBS for 15 min.

Finally, FISH probes were denatured at 80 °C for 5 min and brought together with pre-treated samples in pre-warmed hybridization chambers. After 5 min incubation at 75 °C, hybridization was performed in sealed chambers at 37 °C overnight. Non-hybridized probe was removed by three washing steps in post-hybridization solution (50 % formamide in 2xSSC) at 45 °C for 10 min and two washing steps in 2xSSC at 45 °C for 5 min. Following 20 min incubation in 4 % BSA/2xSSC blocking solution, hybridized probes were detected with Cy5-conjugated streptavidin (1:200 in 2 % BSA/PBS/0.05 % Tween). Signals were further enhanced by streptavidin–biotin (1:250 in 2 % BSA/PBS/0.05 % Tween) detection followed by another Cy5-conjugated streptavidin detection. Finally, DNA counterstain was performed with 4′,6-diamidino-2-phenylindole (DAPI; 200 ng/ml; Sigma-Aldrich, Germany) and samples were mounted using Vectashield antifade mounting medium (Vector Laboratories, USA).

### Microscopy and image analysis

Confocal optical Z stacks of images (*xyz* voxel size: 80 × 80 × 200 nm) were obtained using a Leica SP5 laser scanning microscope, equipped with 63×/1.4 NA oil immersion objective. Fluorophores were excited with 405 nm (for DAPI detection), 488 nm (for Alexa 488 detection) and 633 nm (for Cy5 detection) laser lines. Imaging acquisition parameters were selected carefully to avoid under- and overexposed pixels, while keeping the imaging conditions constant. Distance measurements and analysis were performed as previously described [33]. Nuclear periphery was defined by the edge of the DAPI signal. Constitutive heterochromatin (chromocenters) was identified using the high-intensity DAPI signals and, in the case of the ectopic MeCP2-expressing cells, by anti-MeCP2 antibody immunofluorescence staining.

### Databases and genomic context analysis

Suitable BACs as well as neighboring genes were identified in the “cytoview” display of the Ensembl Genome Browser (http://www.ensembl.org, [62]). For the 2- and 5-Mbp windows, distances of 1 or 2.5 Mbp were calculated upstream and downstream from the center of each gene.

The gene activity of genomic regions was calculated as the average of all Affymetrix probe sets overlapping with the corresponding genomic regions. The number of genes (gene density) and the number of CpG islands were retrieved from the Ref-genes and CpG entries, respectively, in the genome browser (m38 assembly) overlapping with the corresponding genomic coordinates. GC content (fraction of GC within sequence), LINE and SINE density (percentage of covered sequences) were calculated using the corresponding genomic regions submitted to RepeatMasker (http://www.repeatmasker.org, version open-4.0; [63]).

### Statistics and data visualization

Microarray analyses were performed using Affymetrix GeneChip^®^ Operating Software (GCOS) for quality check, RMA-Express 0.3 for normalization and in-house statistical software for further testing (Nalimov test, ANOVA, *t* tests) and descriptive statistic (details provided in the Additional file 1: Table S8). Data analyses of all other measurements were performed using Excel software (Microsoft Cooperation, USA). The fold change of the selected genes was plotted against the –log base 10 of the *p* value of the *t* test calculated for the fold change by GCOS. If multiple Affymetrix probe sets were present for the same gene, the maximum fold change variant was selected. Volcano plots were generated with R open source software (http://www.r-project.org/; [64]). Plot layouts were further processed with Adobe Illustrator (Adobe Systems Incorporated).

### Correlation analysis

All correlation analysis was performed using R and the Stats Package (version 3.2.0). Single gene expression fold changes were analyzed for correlation to the change (Δ *D*) of gene positioning either toward the chromocenter or the nuclear periphery, based on a confidence level of 0.95 by the Pearson’s correlation coefficient (*R*). The correlation between gene expression fold change of whole BACs and the distance to heterochromatin was calculated as described above, using the cumulative gene expression fold change per BAC. The latter was calculated as the average of maximum gene expression fold changes. For genes partially contained within the corresponding BAC, the expression was adjusted to the gene length overlapping with the BAC. For this purpose, the length of the gene overlapping with the BAC was divided by the total gene length and this fraction multiplied by the expression. Correlation coefficients between the features of the 2- and 5-Mbp genomic environment and the normalized distances to the chromocenter and nuclear periphery were calculated as described above using the normalized distances measured in myotubes, myoblasts, and high and low MeCP2-expressing cells, respectively.

## Additional file

**Additional file 1.** Supplementary information figures and tables.

## Abbreviations

3D: three dimensional
ANOVA: analysis of variance
BAC: bacterial artificial chromosome
BSA: bovine serum albumin
DAPI: 4′,6′-diamidino-2-phenylindole
DAVID: Database of Annotation, Visualization And Integrated Discovery
FACS: fluorescent-activated cell sorting
FISH: fluorescence in situ hybridization
GPE: Gaussian propagation error
LINE: long interspersed nuclear element
MB: myoblast
MT: myotube
PBS: phosphate-buffered saline
PEV: position-effect variegation
RIDGE: regions of increased gene expression
SINE: short interspersed nuclear element
SSC: saline–sodium citrate
YFP: yellow fluorescent protein
