# Supplementary material for: Gene repositioning within the cell nucleus is not random and is determined by its genomic neighborhood
Source: Epigenetics Chromatin. 2015 Sep 17;8:36. doi: 10.1186/s13072-015-0025-5 (PMC4574441; doi:10.1186/s13072-015-0025-5)
Supplement: Supplementary file 1 — Additional file 1. Supplementary information figures and tables. [file 13072_2015_25_MOESM1_ESM.pdf]

**Figure S1. Biological validation of expression profiling data.**

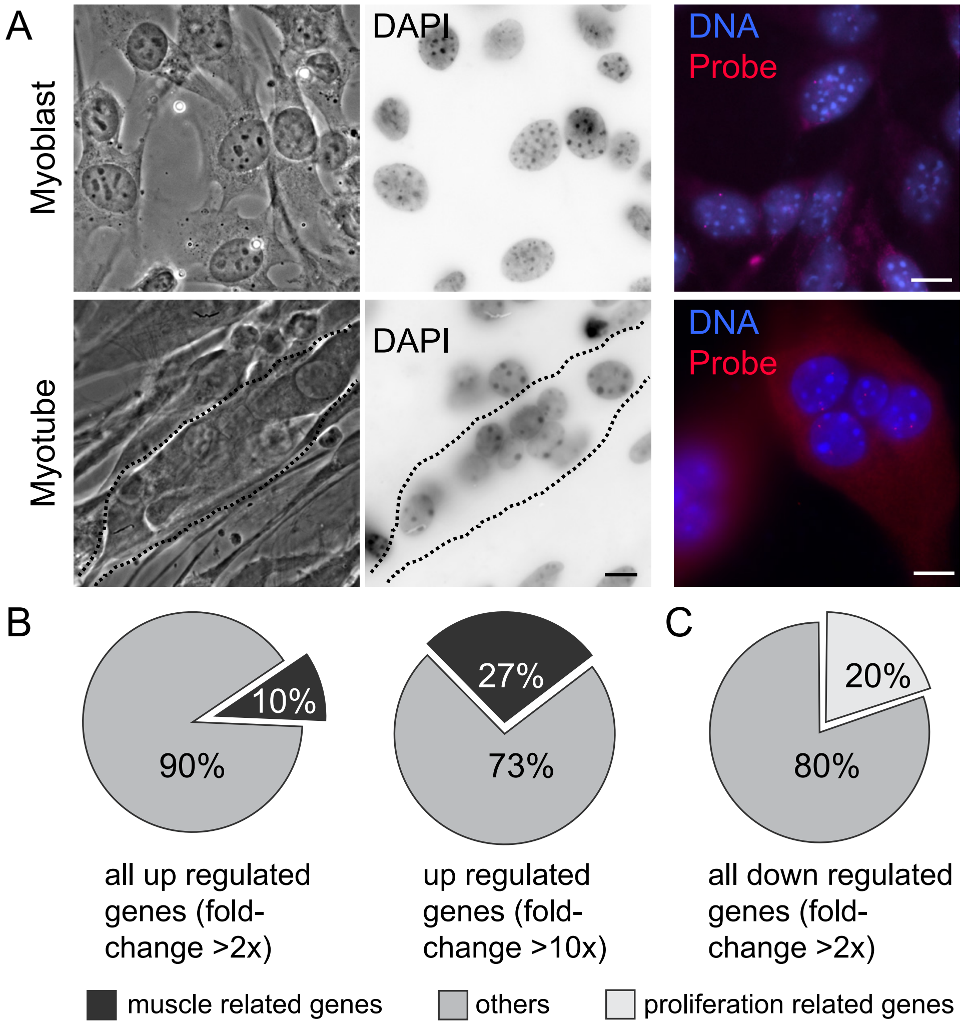


**A** Representatitve images of the morphological changes during myogenic differentiation and tadditional 3D-FISH fluorescent image data with gene locus in red and DNA counterstaining with DAPI (blue) Bar: 10 µm. **B-C** All genes detected in the transcriptional profiling with statistical significance and at least two fold expression (up and down) were analyzed for their gene ontology using the DAVID database. Pie charts (grouped by fold changes) give the ratio of muscle related (dark grey) and proliferation related genes (light grey), respectively.

**Figure S2. Morphological differences in cellular systems.**

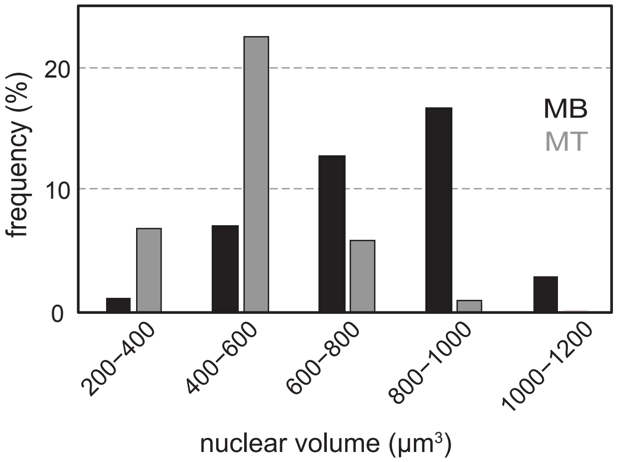


The nuclear volume of >30 nuclei from myoblasts (MB, black) and myotubes (MT, grey) were evaluated, resulting in the shown frequency distribution of volume bins. During myogenesis the nuclear volume decreases to half, which consequently reduces all distances measured. This stark morphological change together with an increased clustering of constitutive heterochromatin, compels the use of a normalization procedure.

**Table S1. 3D-distance measurements in undifferentiated myoblasts (MB) and differentiated myotubes (MT) (differentiation system).**

| **A (diff. sys)** | **3D distances: gene to nuclear periphery** | | | | | | | |
|---|---|---|---|---|---|---|---|---|
| | **Absolute 3D distance [µm]** | | | | **single cell normalized 3D distances** | | | |
| **Gene** | AVG (MT) | AVG (MB) | Δ D [µm] | GPE | AVG n (MT) | AVG n (MB) | Δ nD | GPE |
| *Birc5* | 1.88 (±0.83) | 1.53 (±0,66) | 0.34 | 1.06 | 0.77 (±0.17) | 0.76 (±0.18) | 0.01 | 0.25 |
| *Brca1* | 1.67 (±0.60) | 1.10 (±0.41) | 0.57 | 0.72 | 0.84 (±0.14) | 0.74 (±0.17) | 0.10 | 0.22 |
| *Coro1c* | 1.14 (±0.63) | 1.35 (±0.78) | - 0.21 | 1.00 | 0.58 (±0.20) | 0.67 (±0.24) | - 0.09 | 0.31 |
| *Mef2c* | 1.23 (±0.45) | 1.10 (±0.59) | 0.13 | 0.74 | 0.66 (±0.15) | 0.64 (±0.23) | 0.02 | 0.28 |
| *Myom2* | 0.95 (±0.57) | 0.74 (±0.52) | 0.21 | 0.77 | 0.51 (±0.19) | 0.54 (±0.30) | - 0.03 | 0.35 |
| *Nrde2* | 1.33 (±0.47) | 0.96 (±0.40) | 0.37 | 0.62 | 0.74 (±0.14) | 0.63 (±0.18) | 0.11 | 0.23 |
| *Obscn* | 1.61 (±0.69) | 1.25 (±0.73) | 0.36 | 1.00 | 0.72 (±0.17) | 0.69 (±0.27) | 0.03 | 0.32 |
| *Slc19a2* | 1.87 (±0.73) | 0.91 (±0.48) | 0.96 | 0.88 | 0.75 (±0.16) | 0.63 (±0.23) | 0.12 | 0.28 |
| *Tpm3* | 1.07 (±0.45) | 1.09 (±0.50) | - 0.02 | 0.68 | 0.63 (±0.18) | 0.66 (±0.21) | - 0.03 | 0.27 |
| *Ttk* | 1.59 (±0.84) | 1.07 (±0.78) | 0.51 | 1.15 | 0.68 (±0.21) | 0.58 (±0.27) | 0.11 | 0.34 |
| **B (diff. sys)** | **3D distances: gene to chromocenter** | | | | | | | |
| | **Absolute 3D distance [µm]** | | | | **Single cell normalized 3D distances** | | | |
| Gene | AVG (MT) | AVG (MB) | Δ D [µm] | GPE | AVG n (MT) | AVG n (MB) | Δ nD | GPE |
| *Birc5* | 1.25 (±0.55) | 0.97 (±0,44) | 0.29 | 0.70 | 0.66 (±0.22) | 0.60 (±0.21) | 0.06 | 0.31 |
| *Brca1* | 1.22 (±0.51) | 1.00 (±0.56) | 0.22 | 0.76 | 0.65 (±0.24) | 0.67 (±0.23) | - 0.01 | 0.33 |
| *Coro1c* | 1.38 (±0.62) | 1.19 (±0.59) | 0.20 | 0.86 | 0.66 (±0.23) | 0.63 (±0.25) | 0.03 | 0.34 |
| *Mef2c* | 0.98(±0.57) | 0.94 (±0.56) | 0.22 | 0.83 | 0.58 (±0.23) | 0.55 (±0.22) | 0.04 | 0.31 |
| *Myom2* | 1.04 (±0.52) | 0.97 (±0.44) | 0.01 | 0.72 | 0.50 (±0.26) | 0.58 (±0.21) | - 0.07 | 0.33 |
| *Nrde2* | 1.24 (±0.62) | 1.38 (±0.70) | - 0.34 | 0.88 | 0.60 (±0.25) | 0.72 (±0.23) | - 0.13 | 0.34 |
| *Obscn* | 1.35 (±0.62) | 1.04 (±0.54) | 0.20 | 0.82 | 0.61 (±0.24) | 0.54 (±0.25) | 0.07 | 0.35 |
| *Slc19a2* | 1.21 (±0.60) | 1.23 (±0.60) | 0.12 | 0.86 | 0.65 (±0.22) | 0.72 (±0.22) | - 0.07 | 0.31 |
| *Tpm3* | 1.21 (±0.60) | 1.08 (±0.54) | 0.12 | 0.81 | 0.62 (±0.25) | 0.67 (±0.22) | - 0.05 | 0.33 |
| *Ttk* | 1.01 (±0.56) | 1.11 (±0.46) | - 0.11 | 0.72 | 0.51 (±0.24) | 0.63 (±0.20) | - 0.11 | 0.32 |

Genes selected in the *in vitro* differentiation system were visualized by 3D-FISH using BAC probes and distances towards the nearest heterochromatin compartment indicated were measured and averaged for undifferentiated myoblasts (MB) and differentiated myotubes (MT). Average distances towards the nuclear periphery (A) or towards the nearest chromocenter (B) are given in the respective AVG column with standard deviation included in

brackets (for detailed information see also Table S3 and Figure S5). The average distance change (Δ D) was calculated by subtracting the mean distance values of high and low expressing cells. Linear Gaussian propagation error (GPE) was calculated as the square root of the sum of squared individual standard deviations per sample. Single nucleus normalization (n) was performed as described in the methods yielding comparable distance changes unaffected by nuclear morphology differences. A negative Δ D (red) refers to a decrease in distance or movement towards the compartment during differentiation.

**Table S2. 3D-distance measurements in high and low expressing MeCP2 cells (ectopic heterochromatin reorganization system).**

| **A (ect. sys.)** | **3D distances: gene to nuclear periphery** | | | | | | | |
|---|---|---|---|---|---|---|---|---|
| | **Absolute 3D distance [µm]** | | | | **single cell normalized 3D distances** | | | |
| Gene | High MeCP2 AVG | Low MeCP2 AVG | Δ D [µm] | GPE | High MeCP2 AVG n | Low MeCP2 AVG n | Δ nD | GPE |
| *Bdnf* | 0.76 (±0.38) | 0.74 (±0,45) | 0.02 | 0.58 | 0.54 (±0.20) | 0.54 (±0.23) | 0.00 | 0.30 |
| *Birc5* | 1.69 (±0.65) | 1.49 (±0.80) | 0.20 | 1.04 | 0.79 (±0.18) | 0.72 (±0.21) | 0.06 | 0.28 |
| *Brca1* | 1.72 (±0.74) | 1.70 (±0.64) | 0.02 | 0.98 | 0.79 (±0.18) | 0.81 (±0.16) | - 0.02 | 0.24 |
| *Cdc20* | 0.83 (±0.45) | 0.76 (±0.45) | 0.07 | 0.64 | 0.56 (±0.21) | 0.53 (±0.25) | 0.02 | 0.32 |
| *Col6a2* | 1.30 (±0.57) | 1.04 (±0.49) | 0.26 | 0.75 | 0.72 (±0.16) | 0.67 (±0.20) | 0.05 | 0.26 |
| *Myom2* | 1.00 (±0.69) | 0.99 (±0.63) | 0.00 | 0.93 | 0.57(±0.25) | 0.58 (±0.63) | 0.00 | 0.35 |
| *Prl7c1* | 1.38 (±0.75) | 1.21 (±0.64) | 0.16 | 0.98 | 0.71 (±0.24) | 0.67 (±0.24) | 0.04 | 0.34 |
| *Ttk* | 0.92 (±0.46) | 0.82 (±0.53) | 0.10 | 0.71 | 0.60 (±0.22) | 0.56 (±0.25) | 0.04 | 0.33 |

| **B (ect. Sys.)** | **3D distances: gene to chromocenter** | | | | | | | |
|---|---|---|---|---|---|---|---|---|
| | **Absolute 3D distance [µm]** | | | | **Single cell normalized 3D distances** | | | |
| Gene | High MeCP2 AVG | Low MeCP2 AVG | Δ D [µm] | GPE | High MeCP2 AVG n | Low MeCP2 AVG n | Δ nD | GPE |
| *Bdnf* | 1.47 (±0.79) | 0.99 (±0,52) | 0.48 | 0.94 | 0.72 (±0.25) | 0.68 (±0.25) | 0.00 | 0.36 |
| *Birc5* | 0.89 (±0.51) | 0.94 (±0.49) | - 0.04 | 0.70 | 0.50(±0.22) | 0.60 (±0.24) | - 0.09 | 0.32 |
| *Brca1* | 0.93 (±0.45) | 0.86 (±0.51) | 0.07 | 0.68 | 0.56 (±0.22) | 0.54 (±0.26) | 0.02 | 0.34 |
| *Cdc20* | 1.36 (±0.65) | 1.04 (±0.59) | 0.32 | 0.88 | 0.72 (±0.23) | 0.69 (±0.21) | 0.03 | 0.31 |
| *Col6a2* | 0.94 (±0.56) | 0.78 (±0.47) | 0.17 | 0.73 | 0.52 (±0.23) | 0.59 (±0.24) | - 0.07 | 0.33 |
| *Myom2* | 0.86 (±0.48) | 0.71 (±0.38) | 0.15 | 0.62 | 0.57 (±0.25) | 0.52 (±0.24) | 0.05 | 0.34 |
| *Prl7c1* | 0.68 (±0.38) | 0.69 (±0.39) | - 0.01 | 0.55 | 0.47 (±0.22) | 0.50 (±0.23) | - 0.03 | 0.32 |
| *Ttk* | 1.05 (±0.61) | 0.85 (±0.53) | 0.20 | 0.81 | 0.58 (±0.25) | 0.59 (±0.28) | - 0.01 | 0.37 |

Genes selected in the ectopic MeCP2 system were visualized by 3D-FISH using BAC probes and distances towards the nearest heterochromatin compartment were measured and averaged for high and low MeCP2 expressing cells. Average distances towards the nuclear periphery (A) or towards nearest chromocenter (B) are given in the respective AVG column with standard deviation included in brackets (for detailed information see also Table S4 and Figure S6). The average distance change (Δ D) was calculated by subtracting the mean distance values of high and low expressing cells. Linear Gaussian propagation error (GPE) was calculated as the square root of the sum of squared individual standard deviations per sample. Single nucleus normalization (n) was performed as described in the methods yielding comparable distance changes. Negative Δ D values (red) refer to a decrease in distance or movement towards the indicated compartment in differentiation independent reorganized heterochromatin (i.e. high levels of MeCP2).

**Figure S3. Average normalized 3D distances per gene and condition towards the heterochromatin compartment indicated.**

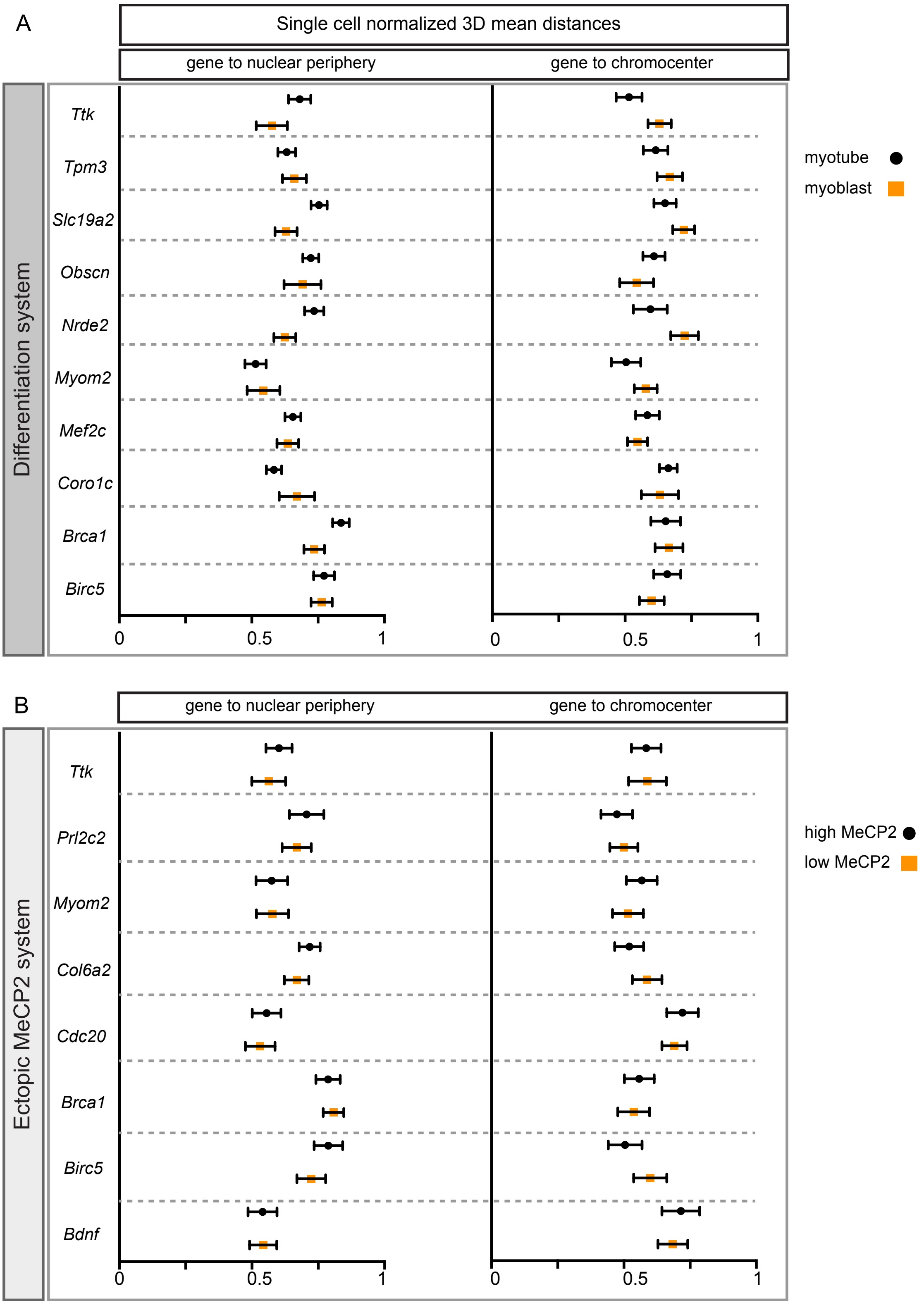


Overview of normalized 3D distance measurements (see Tables S1 and S2) in the differentiation system (A) and differentiation independent ectopic MeCP2 system (B). Means are highlighted either as black circles (myotubes and high MeCP2 levels) or orange squares (myoblasts and low MeCP2 levels); whiskers indicate the 95% confidence interval.

**Figure S4. Average normalized distance changes per gene and condition towards the heterochromatin compartment indicated.**

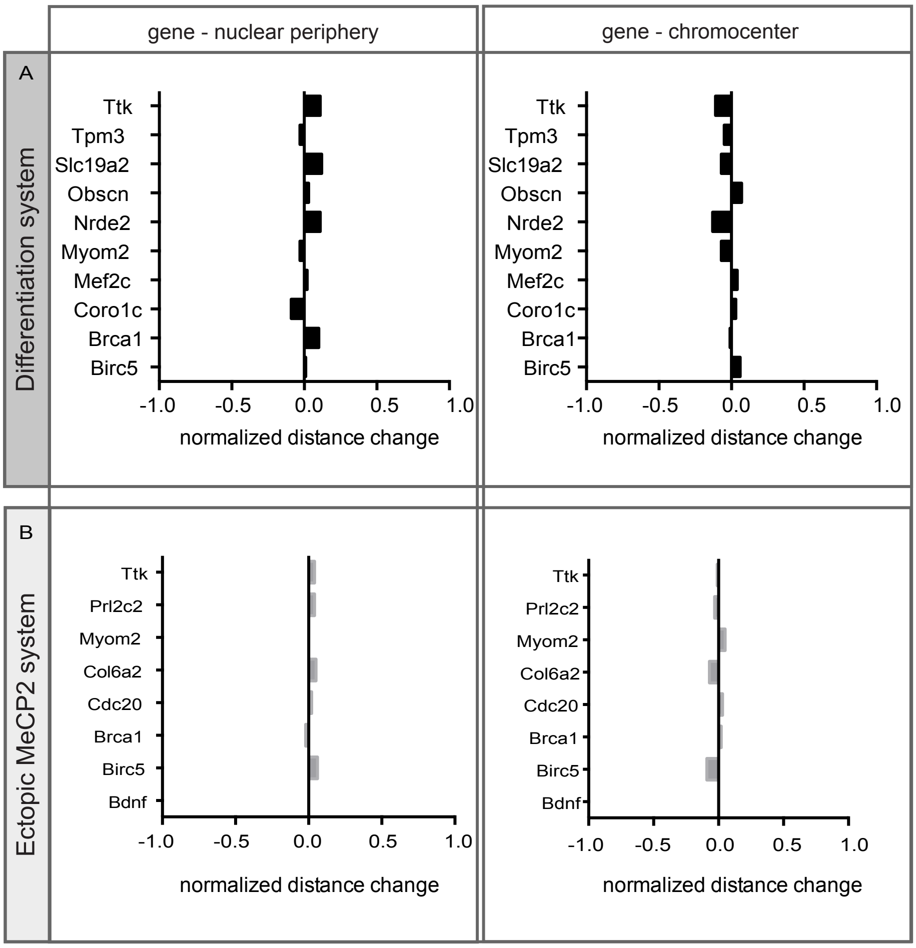


Overview of normalized 3D distance changes (see Tables S1 and S2) in the differentiation system (A) and differentiation independent ectopic MeCP2 system (B).

**Table S3. A) Statistics summary table of 3D distance measurements (differentiation system).**

| | Gene | *Birc5* | | *Brca1* | | *Coro1c* | | *Mef2c* | | *Myom2* | | *Nrde2* | | *Obscn* | | *Slc19a2* | | *Tpm3* | | *Ttk* | |
|---|---|---|---|---|---|---|---|---|---|---|---|---|---|---|---|---|---|---|---|---|---|
| A | Dataset | MT | MB | MT | MB | MT | MB | MT | MB | MT | MB | MT | MB | MT | MB | MT | MB | MT | MB | MT | MB |
| | Sample size | 77 | 80 | 74 | 76 | 188 | 51 | 100 | 126 | 86 | 92 | 61 | 77 | 129 | 61 | 111 | 116 | 113 | 84 | 100 | 83 |
| abs. distances [µm];gene to chromocenter | AVG | 1.25 | 0.97 | 1.22 | 1.00 | 1.38 | 1.19 | 1.16 | 0.94 | 0.98 | 0.97 | 1.04 | 1.38 | 1.24 | 1.04 | 1.35 | 1.23 | 1.21 | 1.08 | 1.01 | 1.11 |
| | STD | 0.55 | 0.44 | 0.51 | 0.56 | 0.62 | 0.59 | 0.61 | 0.56 | 0.57 | 0.44 | 0.52 | 0.70 | 0.62 | 0.54 | 0.62 | 0.60 | 0.60 | 0.54 | 0.56 | 0.46 |
| | Q0 | 0.18 | 0.25 | 0.08 | 0.00 | 0.00 | 0.16 | 0.00 | 0.00 | 0.00 | 0.23 | 0.00 | 0.00 | 0.00 | 0.19 | 0.18 | 0.00 | 0.00 | 0.11 | 0.16 | 0.00 |
| | Q1 | 0.91 | 0.65 | 0.89 | 0.58 | 0.99 | 0.75 | 0.79 | 0.61 | 0.52 | 0.67 | 0.74 | 0.88 | 0.82 | 0.62 | 0.94 | 0.82 | 0.78 | 0.71 | 0.59 | 0.80 |
| | Q2 | 1.18 | 0.92 | 1.19 | 1.00 | 1.35 | 1.18 | 1.07 | 0.82 | 0.89 | 0.87 | 1.03 | 1.28 | 1.18 | 1.03 | 1.23 | 1.17 | 1.20 | 1.03 | 0.92 | 1.09 |
| | Q3 | 1.63 | 1.24 | 1.53 | 1.20 | 1.71 | 1.67 | 1.46 | 1.15 | 1.38 | 1.17 | 1.39 | 1.88 | 1.56 | 1.38 | 1.74 | 1.62 | 1.58 | 1.38 | 1.34 | 1.39 |
| | Q4 | 2.99 | 2.22 | 2.52 | 3.15 | 3.31 | 2.40 | 3.84 | 3.46 | 2.36 | 2.28 | 2.21 | 3.32 | 3.17 | 2.37 | 3.71 | 3.21 | 3.04 | 2.49 | 3.05 | 2.14 |
| | SEM | 0.06 | 0.05 | 0.06 | 0.06 | 0.05 | 0.30 | 0.06 | 0.19 | 0.06 | 0.22 | 0.07 | 0.08 | 0.05 | 0.07 | 0.06 | 0.26 | 0.06 | 0.23 | 0.06 | 0.05 |
| | Δ D | 0.29 | | 0.22 | | 0.20 | | 0.22 | | 0.01 | | -0.34 | | 0.20 | | 0.12 | | 0.12 | | -0.11 | |
| | GPE | 0.70 | | 0.76 | | 0.86 | | 0.83 | | 0.72 | | 0.88 | | 0.82 | | 0.86 | | 0.81 | | 0.72 | |
| single nucleus normalized distances | AVG | 0.66 | 0.60 | 0.65 | 0.67 | 0.66 | 0.63 | 0.58 | 0.55 | 0.50 | 0.58 | 0.60 | 0.72 | 0.61 | 0.54 | 0.65 | 0.72 | 0.62 | 0.67 | 0.51 | 0.63 |
| | STD | 0.22 | 0.21 | 0.24 | 0.23 | 0.23 | 0.25 | 0.23 | 0.22 | 0.26 | 0.21 | 0.25 | 0.23 | 0.24 | 0.25 | 0.22 | 0.22 | 0.25 | 0.22 | 0.24 | 0.20 |
| | Q0 | 0.16 | 0.17 | 0.08 | 0.09 | 0.07 | 0.13 | 0.05 | 0.08 | 0.07 | 0.13 | 0.07 | 0.10 | 0.07 | 0.13 | 0.13 | 0.07 | 0.07 | 0.16 | 0.10 | 0.07 |
| | Q1 | 0.53 | 0.44 | 0.49 | 0.54 | 0.51 | 0.46 | 0.41 | 0.41 | 0.28 | 0.45 | 0.45 | 0.59 | 0.44 | 0.33 | 0.51 | 0.60 | 0.41 | 0.51 | 0.29 | 0.45 |
| | Q2 | 0.68 | 0.60 | 0.68 | 0.71 | 0.69 | 0.62 | 0.58 | 0.54 | 0.46 | 0.55 | 0.64 | 0.78 | 0.64 | 0.56 | 0.67 | 0.79 | 0.65 | 0.70 | 0.50 | 0.65 |
| | Q3 | 0.87 | 0.78 | 0.86 | 0.86 | 0.86 | 0.88 | 0.78 | 0.72 | 0.75 | 0.69 | 0.81 | 0.90 | 0.80 | 0.77 | 0.85 | 0.90 | 0.81 | 0.85 | 0.71 | 0.80 |
| | Q4 | 1.00 | 0.97 | 1.00 | 0.98 | 0.99 | 0.97 | 1.00 | 1.00 | 0.97 | 0.99 | 0.98 | 1.00 | 1.00 | 0.95 | 0.99 | 1.00 | 0.99 | 0.98 | 0.99 | 0.97 |
| | SEM | 0.03 | 0.02 | 0.03 | 0.03 | 0.02 | 0.13 | 0.02 | 0.08 | 0.03 | 0.10 | 0.03 | 0.03 | 0.02 | 0.03 | 0.02 | 0.09 | 0.02 | 0.09 | 0.02 | 0.02 |
| | Δ D | 0.06 | | -0.01 | | 0.03 | | 0.04 | | -0.07 | | -0.13 | | 0.07 | | -0.07 | | -0.05 | | -0.11 | |
| | GPE | 0.31 | | 0.33 | | 0.34 | | 0.31 | | 0.33 | | 0.34 | | 0.35 | | 0.31 | | 0.33 | | 0.32 | |

**Table S3. B) Statistics summary table of 3D distance measurements (differentiation system).**

| B | Gene | *Birc5* | | *Brca1* | | *Coro1c* | | *Mef2c* | | *Myom2* | | *Nrde2* | | *Obscn* | | *Slc19a2* | | *Tpm3* | | *Ttk* | |
|---|---|---|---|---|---|---|---|---|---|---|---|---|---|---|---|---|---|---|---|---|---|
| | Dataset | MT | MB | MT | MB | MT | MB | MT | MB | MT | MB | MT | MB | MT | MB | MT | MB | MT | MB | MT | MB |
| | Sample size | 77 | 80 | 74 | 76 | 188 | 51 | 100 | 126 | 86 | 92 | 61 | 77 | 129 | 61 | 111 | 116 | 113 | 84 | 100 | 83 |
| abs. distances [µm]; gene to nuc. periphery | AVG | 1.88 | 1.53 | 1.67 | 1.10 | 1.14 | 1.35 | 1.23 | 1.10 | 0.95 | 0.74 | 1.33 | 0.96 | 1.61 | 1.25 | 1.87 | 0.91 | 1.07 | 1.09 | 1.59 | 1.07 |
| | STD | 0.83 | 0.66 | 0.60 | 0.41 | 0.63 | 0.78 | 0.45 | 0.59 | 0.57 | 0.52 | 0.47 | 0.40 | 0.69 | 0.73 | 0.73 | 0.48 | 0.45 | 0.50 | 0.84 | 0.78 |
| | Q0 | 0.30 | 0.35 | 0.45 | 0.29 | 0.08 | 0.00 | 0.21 | 0.00 | 0.20 | 0.00 | 0.50 | 0.30 | 0.32 | 0.00 | 0.43 | 0.00 | 0.16 | 0.00 | 0.16 | 0.00 |
| | Q1 | 1.31 | 1.16 | 1.27 | 0.79 | 0.62 | 0.78 | 0.89 | 0.62 | 0.54 | 0.21 | 0.98 | 0.60 | 1.09 | 0.65 | 1.26 | 0.58 | 0.82 | 0.76 | 1.01 | 0.43 |
| | Q2 | 1.73 | 1.51 | 1.64 | 1.04 | 1.06 | 1.24 | 1.23 | 1.08 | 0.76 | 0.76 | 1.32 | 0.97 | 1.57 | 1.18 | 1.81 | 0.79 | 0.99 | 1.07 | 1.38 | 0.97 |
| | Q3 | 2.38 | 1.93 | 2.12 | 1.37 | 1.53 | 1.81 | 1.57 | 1.51 | 1.37 | 1.13 | 1.63 | 1.25 | 2.06 | 1.94 | 2.37 | 1.17 | 1.33 | 1.43 | 2.09 | 1.46 |
| | Q4 | 4.08 | 3.69 | 3.06 | 2.19 | 3.35 | 3.54 | 2.15 | 2.56 | 3.01 | 2.14 | 2.43 | 2.10 | 3.50 | 2.57 | 3.69 | 2.57 | 2.19 | 2.54 | 3.75 | 4.13 |
| | SEM | 0.09 | 0.07 | 0.07 | 0.05 | 0.05 | 0.36 | 0.05 | 0.20 | 0.06 | 0.16 | 0.06 | 0.05 | 0.06 | 0.09 | 0.07 | 0.18 | 0.04 | 0.22 | 0.08 | 0.02 |
| | Δ D | 0.34 | | 0.57 | | -0.21 | | 0.13 | | 0.21 | | 0.37 | | 0.36 | | 0.96 | | -0.02 | | 0.51 | |
| | GPE | 1.06 | | 0.72 | | 1.00 | | 0.74 | | 0.77 | | 0.62 | | 1.00 | | 0.88 | | 0.68 | | 1.15 | |
| single nucleus normalized distances | AVG | 0.77 | 0.76 | 0.84 | 0.74 | 0.58 | 0.67 | 0.66 | 0.64 | 0.51 | 0.54 | 0.74 | 0.63 | 0.72 | 0.69 | 0.75 | 0.63 | 0.63 | 0.66 | 0.68 | 0.58 |
| | STD | 0.17 | 0.18 | 0.14 | 0.17 | 0.20 | 0.24 | 0.15 | 0.23 | 0.19 | 0.30 | 0.14 | 0.18 | 0.17 | 0.27 | 0.16 | 0.23 | 0.18 | 0.21 | 0.21 | 0.27 |
| | Q0 | 0.28 | 0.34 | 0.43 | 0.32 | 0.15 | 0.11 | 0.22 | 0.05 | 0.20 | 0.03 | 0.45 | 0.26 | 0.28 | 0.05 | 0.31 | 0.06 | 0.17 | 0.04 | 0.18 | 0.05 |
| | Q1 | 0.67 | 0.64 | 0.75 | 0.61 | 0.42 | 0.52 | 0.55 | 0.47 | 0.37 | 0.23 | 0.63 | 0.48 | 0.60 | 0.51 | 0.62 | 0.50 | 0.55 | 0.54 | 0.54 | 0.36 |
| | Q2 | 0.81 | 0.80 | 0.86 | 0.76 | 0.60 | 0.73 | 0.68 | 0.68 | 0.46 | 0.62 | 0.73 | 0.61 | 0.73 | 0.74 | 0.78 | 0.64 | 0.63 | 0.66 | 0.70 | 0.60 |
| | Q3 | 0.91 | 0.91 | 0.95 | 0.87 | 0.74 | 0.87 | 0.78 | 0.83 | 0.66 | 0.81 | 0.85 | 0.79 | 0.87 | 0.94 | 0.89 | 0.83 | 0.77 | 0.82 | 0.86 | 0.80 |
| | Q4 | 1.00 | 1.00 | 1.00 | 1.00 | 0.99 | 1.00 | 0.89 | 0.99 | 0.97 | 1.00 | 0.98 | 1.00 | 1.00 | 1.00 | 0.99 | 1.00 | 0.95 | 1.00 | 1.00 | 1.00 |
| | SEM | 0.02 | 0.02 | 0.02 | 0.02 | 0.01 | 0.14 | 0.02 | 0.09 | 0.02 | 0.10 | 0.03 | 0.02 | 0.02 | 0.03 | 0.02 | 0.09 | 0.02 | 0.10 | 0.02 | 0.03 |
| | Δ D | 0.01 | | 0.10 | | -0.09 | | 0.02 | | -0.03 | | 0.11 | | 0.03 | | 0.12 | | -0.03 | | 0.11 | |
| | GPE | 0.25 | | 0.22 | | 0.31 | | 0.28 | | 0.35 | | 0.23 | | 0.32 | | 0.28 | | 0.27 | | 0.34 | |

Detailed statistical overview of 3D distance measurements within the differentiation system. (A) Addresses distances and distance changes Δ D towards chromocenters (B) towards the nuclear periphery. “Data set” refers to the respective chromatin state (i.e., either myoblasts [MB] or myotubes [MT]). The total number of measurements is indicated as sample size. For each data set absolute and normalized values are given as: mean distances (AVG), standard deviation (STD), min. (Q0), max. (Q4), median (Q2), standard error of the mean (SEM), and mean distance changes (Δ D) (see also Figure S5). Δ D was calculated as the difference of mean MT and mean MB distances. The linear Gaussian propagation error (GPE) was calculated as the square root of the sum of squared individual standard deviations per sample.

**Table S4. A) Statistics summary table of 3D distance measurements (ectopic MeCP2 system).**

| A | Gene | *Bdnf* | | *Birc5* | | *Brca1* | | *Cdc20* | | *Col6a2* | | *Myom2* | | *Prl2c2* | | *Ttk* | |
|---|---|---|---|---|---|---|---|---|---|---|---|---|---|---|---|---|---|
| | MeCP2 level | high | low | high | low | high | low | high | low | high | low | high | low | high | low | high | low |
| | Sample size | 54 | 77 | 47 | 59 | 62 | 72 | 61 | 77 | 68 | 74 | 72 | 65 | 56 | 77 | 78 | 61 |
| absolute distances [µm] gene to chromocenter | AVG | 1.47 | 0.99 | 0.89 | 0.94 | 0.93 | 0.86 | 1.36 | 1.04 | 0.94 | 0.78 | 0.86 | 0.71 | 0.68 | 0.69 | 1.05 | 0.85 |
| | STD | 0.79 | 0.52 | 0.51 | 0.49 | 0.45 | 0.51 | 0.65 | 0.59 | 0.56 | 0.47 | 0.48 | 0.38 | 0.38 | 0.39 | 0.61 | 0.53 |
| | Q0 | 0.00 | 0.25 | 0.00 | 0.08 | 0.00 | 0.00 | 0.08 | 0.00 | 0.08 | 0.00 | 0.08 | 0.00 | 0.00 | 0.00 | 0.00 | 0.00 |
| | Q1 | 0.94 | 0.57 | 0.54 | 0.56 | 0.66 | 0.47 | 0.82 | 0.69 | 0.56 | 0.48 | 0.47 | 0.41 | 0.42 | 0.43 | 0.61 | 0.43 |
| | Q2 | 1.45 | 0.94 | 0.80 | 0.90 | 0.89 | 0.77 | 1.20 | 0.88 | 0.80 | 0.69 | 0.77 | 0.67 | 0.61 | 0.66 | 0.92 | 0.88 |
| | Q3 | 2.01 | 1.28 | 1.11 | 1.19 | 1.17 | 1.26 | 1.91 | 1.27 | 1.21 | 1.01 | 1.22 | 0.92 | 0.98 | 0.94 | 1.40 | 1.24 |
| | Q4 | 3.09 | 2.53 | 2.44 | 2.08 | 2.01 | 2.20 | 2.46 | 4.03 | 2.88 | 2.62 | 2.57 | 1.89 | 1.64 | 1.70 | 2.77 | 2.36 |
| | SEM | 0.11 | 0.06 | 0.07 | 0.06 | 0.06 | 0.06 | 0.08 | 0.07 | 0.07 | 0.06 | 0.06 | 0.05 | 0.05 | 0.04 | 0.07 | 0.07 |
| | Δ D | 0.48 | | -0.04 | | 0.07 | | 0.32 | | 0.17 | | 0.15 | | -0.01 | | 0.20 | |
| | GPE | 0.94 | | 0.70 | | 0.68 | | 0.88 | | 0.73 | | 0.62 | | 0.55 | | 0.81 | |
| single nucleus normalized distances | AVG | 0.72 | 0.68 | 0.50 | 0.60 | 0.56 | 0.54 | 0.72 | 0.69 | 0.52 | 0.59 | 0.57 | 0.52 | 0.47 | 0.50 | 0.58 | 0.59 |
| | STD | 0.26 | 0.25 | 0.22 | 0.24 | 0.22 | 0.26 | 0.23 | 0.21 | 0.23 | 0.24 | 0.25 | 0.24 | 0.22 | 0.23 | 0.25 | 0.28 |
| | Q0 | 0.09 | 0.23 | 0.10 | 0.13 | 0.09 | 0.08 | 0.11 | 0.09 | 0.13 | 0.09 | 0.12 | 0.08 | 0.07 | 0.05 | 0.09 | 0.07 |
| | Q1 | 0.54 | 0.43 | 0.34 | 0.43 | 0.41 | 0.32 | 0.55 | 0.55 | 0.35 | 0.41 | 0.33 | 0.32 | 0.31 | 0.33 | 0.37 | 0.37 |
| | Q2 | 0.80 | 0.75 | 0.45 | 0.58 | 0.57 | 0.48 | 0.77 | 0.69 | 0.49 | 0.61 | 0.57 | 0.53 | 0.47 | 0.49 | 0.60 | 0.66 |
| | Q3 | 0.94 | 0.90 | 0.71 | 0.80 | 0.71 | 0.76 | 0.94 | 0.89 | 0.69 | 0.77 | 0.79 | 0.70 | 0.62 | 0.69 | 0.78 | 0.84 |
| | Q4 | 1.00 | 0.99 | 0.97 | 0.97 | 0.96 | 0.95 | 0.99 | 1.00 | 0.99 | 1.00 | 0.98 | 0.95 | 0.91 | 0.92 | 1.00 | 0.98 |
| | SEM | 0.04 | 0.03 | 0.03 | 0.03 | 0.03 | 0.03 | 0.03 | 0.02 | 0.03 | 0.03 | 0.03 | 0.03 | 0.03 | 0.03 | 0.03 | 0.04 |
| | Δ D | 0.03 | | -0.09 | | 0.02 | | 0.03 | | -0.07 | | 0.05 | | -0.03 | | -0.01 | |
| | GPE | 0.36 | | 0.32 | | 0.34 | | 0.31 | | 0.33 | | 0.34 | | 0.32 | | 0.37 | |

**Table S4. B) Statistics summary table of 3D distance measurements (ectopic MeCP2 system).**

| B | Gene | *Bdnf* | | *Birc5* | | *Brca1* | | *Cdc20* | | *Col6a2* | | *Myom2* | | *Prl2c2* | | *Ttk* | |
|---|---|---|---|---|---|---|---|---|---|---|---|---|---|---|---|---|---|
| | MeCP2 level | high | low | high | low | high | low | high | low | high | low | high | low | high | low | high | low |
| | Sample size | 54 | 77 | 47 | 59 | 62 | 72 | 61 | 77 | 68 | 74 | 72 | 65 | 56 | 77 | 78 | 61 |
| absolute distances [µm] gene to nuclear periphery | AVG | 0.76 | 0.74 | 1.69 | 1.49 | 1.72 | 1.70 | 0.83 | 0.76 | 1.30 | 1.04 | 1.00 | 0.99 | 1.38 | 1.21 | 0.92 | 0.82 |
| | STD | 0.38 | 0.45 | 0.65 | 0.80 | 0.74 | 0.64 | 0.45 | 0.45 | 0.57 | 0.49 | 0.69 | 0.63 | 0.75 | 0.64 | 0.46 | 0.53 |
| | Q0 | 0.16 | 0.00 | 0.08 | 0.19 | 0.08 | 0.16 | 0.21 | 0.00 | 0.20 | 0.20 | 0.00 | 0.00 | 0.11 | 0.08 | 0.00 | 0.00 |
| | Q1 | 0.41 | 0.40 | 1.22 | 0.97 | 1.17 | 1.27 | 0.45 | 0.39 | 0.94 | 0.64 | 0.48 | 0.47 | 0.78 | 0.63 | 0.56 | 0.43 |
| | Q2 | 0.78 | 0.64 | 1.71 | 1.36 | 1.72 | 1.60 | 0.78 | 0.79 | 1.18 | 0.99 | 0.80 | 0.81 | 1.48 | 1.37 | 0.87 | 0.67 |
| | Q3 | 1.03 | 1.04 | 2.21 | 2.04 | 2.23 | 2.14 | 1.08 | 1.17 | 1.63 | 1.39 | 1.36 | 1.38 | 1.80 | 1.76 | 1.35 | 1.23 |
| | Q4 | 1.48 | 1.99 | 2.92 | 3.53 | 3.34 | 3.31 | 2.40 | 1.77 | 3.30 | 2.15 | 2.99 | 2.73 | 3.35 | 2.43 | 1.78 | 2.32 |
| | SEM | 0.05 | 0.05 | 0.10 | 0.10 | 0.09 | 0.08 | 0.06 | 0.05 | 0.07 | 0.06 | 0.08 | 0.08 | 0.10 | 0.07 | 0.05 | 0.07 |
| | Δ D | 0.02 | | 0.20 | | 0.02 | | 0.07 | | 0.26 | | 0.00 | | 0.16 | | 0.10 | |
| | GPE | 0.58 | | 1.04 | | 0.98 | | 0.64 | | 0.75 | | 0.93 | | 0.98 | | 0.71 | |
| single nucleus normalized distances | AVG | 0.54 | 0.54 | 0.79 | 0.72 | 0.79 | 0.81 | 0.56 | 0.53 | 0.72 | 0.67 | 0.57 | 0.58 | 0.71 | 0.67 | 0.60 | 0.56 |
| | STD | 0.20 | 0.23 | 0.18 | 0.21 | 0.18 | 0.16 | 0.21 | 0.25 | 0.16 | 0.20 | 0.25 | 0.24 | 0.24 | 0.24 | 0.22 | 0.25 |
| | Q0 | 0.20 | 0.04 | 0.13 | 0.18 | 0.12 | 0.18 | 0.24 | 0.04 | 0.21 | 0.23 | 0.02 | 0.03 | 0.15 | 0.14 | 0.03 | 0.04 |
| | Q1 | 0.41 | 0.39 | 0.69 | 0.62 | 0.72 | 0.75 | 0.35 | 0.31 | 0.61 | 0.50 | 0.37 | 0.35 | 0.54 | 0.50 | 0.44 | 0.36 |
| | Q2 | 0.52 | 0.51 | 0.84 | 0.77 | 0.83 | 0.83 | 0.56 | 0.58 | 0.72 | 0.68 | 0.53 | 0.55 | 0.77 | 0.76 | 0.62 | 0.53 |
| | Q3 | 0.72 | 0.75 | 0.92 | 0.90 | 0.92 | 0.94 | 0.72 | 0.75 | 0.84 | 0.84 | 0.83 | 0.76 | 0.89 | 0.86 | 0.77 | 0.79 |
| | Q4 | 0.90 | 0.98 | 1.00 | 1.00 | 1.00 | 1.00 | 0.98 | 0.98 | 0.98 | 1.00 | 1.00 | 0.99 | 0.99 | 1.00 | 0.98 | 0.99 |
| | SEM | 0.03 | 0.03 | 0.03 | 0.03 | 0.02 | 0.02 | 0.03 | 0.03 | 0.02 | 0.02 | 0.03 | 0.03 | 0.03 | 0.03 | 0.02 | 0.03 |
| | Δ D | 0.00 | | 0.06 | | -0.02 | | 0.02 | | 0.05 | | 0.00 | | 0.04 | | 0.04 | |
| | GPE | 0.30 | | 0.28 | | 0.24 | | 0.32 | | 0.26 | | 0.35 | | 0.34 | | 0.33 | |

Detailed statistical overview of 3D distance measurements within the ectopic MeCP2 expression system. (A) Addresses distances and distance changes Δ D towards chromocenters (B) towards the nuclear periphery. “Data set” refers to the respective chromatin state (i.e. either high or low MeCP2 expression). The total number of measurements is indicated as sample size. For each data set are given absolute and normalized values as: mean distances (AVG), standard deviation (STD), min. (Q0), max. (Q4), median (Q2), standard error of the mean (SEM), and mean distance changes (Δ D) (see Figure S6). Δ D was calculated as the difference of mean “high MeCP2” and mean “low MeCP2” distances. The linear Gaussian propagation error (GPE) was calculated as the square root of the sum of squared individual standard deviations per sample.

**Figure S5. Tukey box plots of absolute and normalized 3D distances in the differentiation system.**

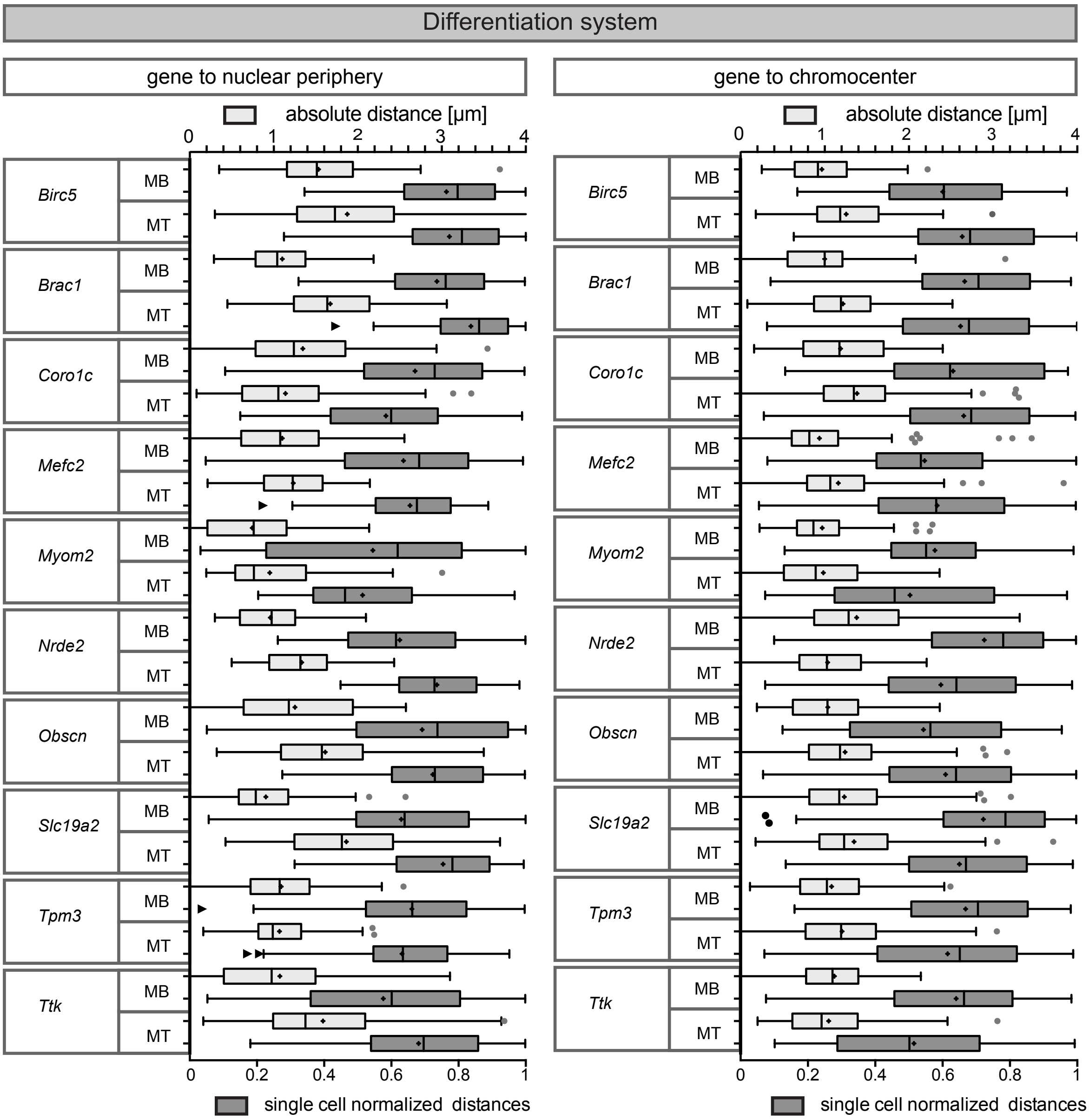


For each observed gene the nuclear position was visualized by 3D-FISH using BAC probes in myoblasts (MB) and myotubes (MT). 3D distance measurements were performed as previously described in methods. The distribution of absolute distances (in µm) towards the nuclear periphery (left) and the nearest chromocenter (right) are shown in light grey (upper x-axis). The distribution of measurements normalized against individually simulated background distributions per nucleus (single cell normalized distances) is shown in dark grey (lower x-axis). Outliers are indicated as dots or triangles; sample means are indicated by crosses and median values are indicated by vertical lines within boxes.

**Figure S6. Tukey box plots of absolute and normalized 3D distances in the ectopic MeCP2 system.**

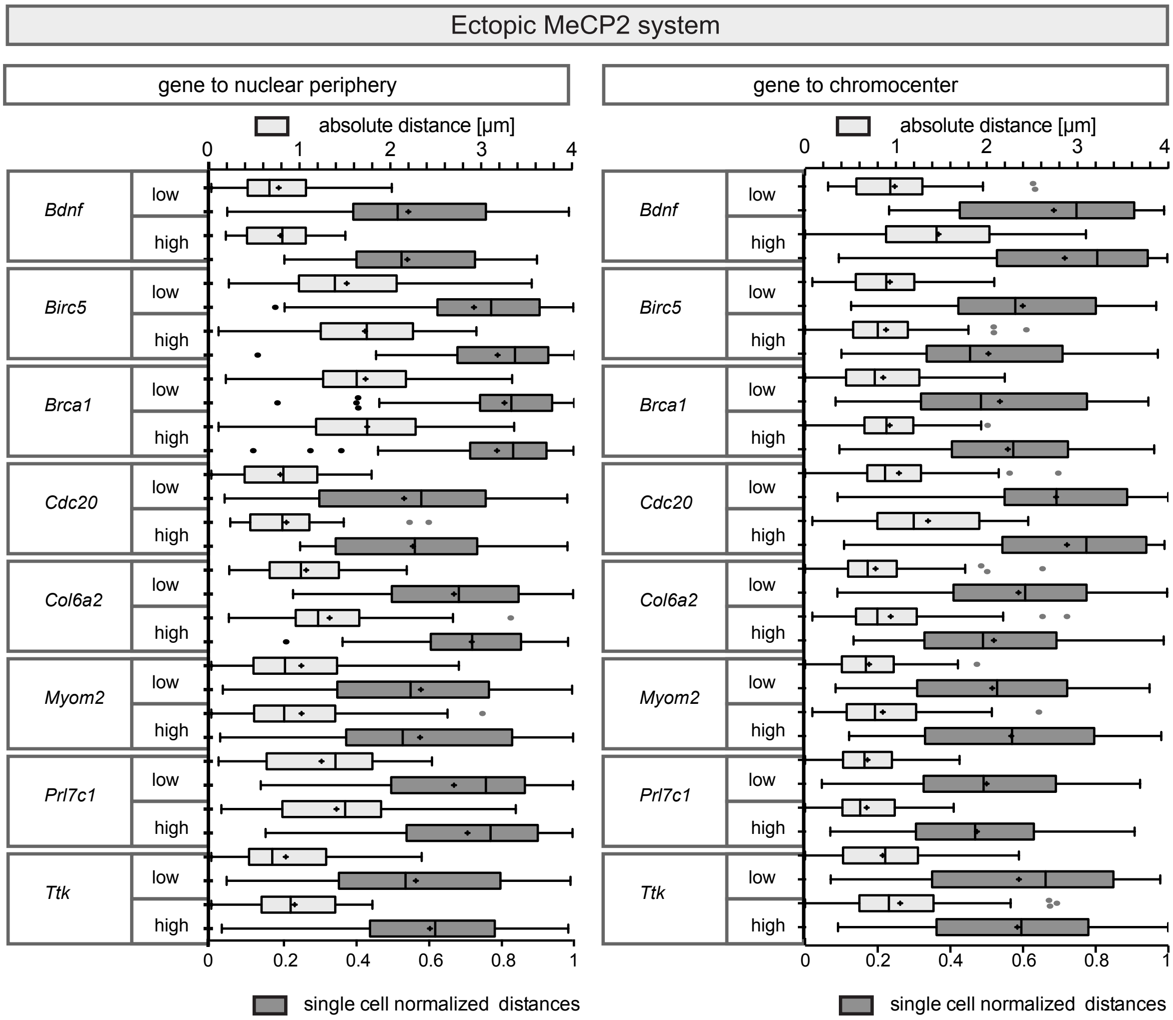


For each observed gene the nuclear position was visualized by 3D-FISH using BAC probes in high and low MeCP2 expressing cells. 3D distance measurements were performed as previously described in methods. The distribution of absolute distances (in µm) towards the nuclear periphery (left) and the nearest chromocenter (right) are shown in light grey (upper x-axis). The distribution of measurements normalized against individually simulated background distributions per nucleus (single cell normalized distances) is shown in dark grey (lower x-axis). Outliers are indicated as dots; sample means are indicated by crosses and median values are indicated by vertical lines within boxes.

**Figure S7. Graphical summary of BAC clone composition, its genomic region and maximum gene expression fold changes.**

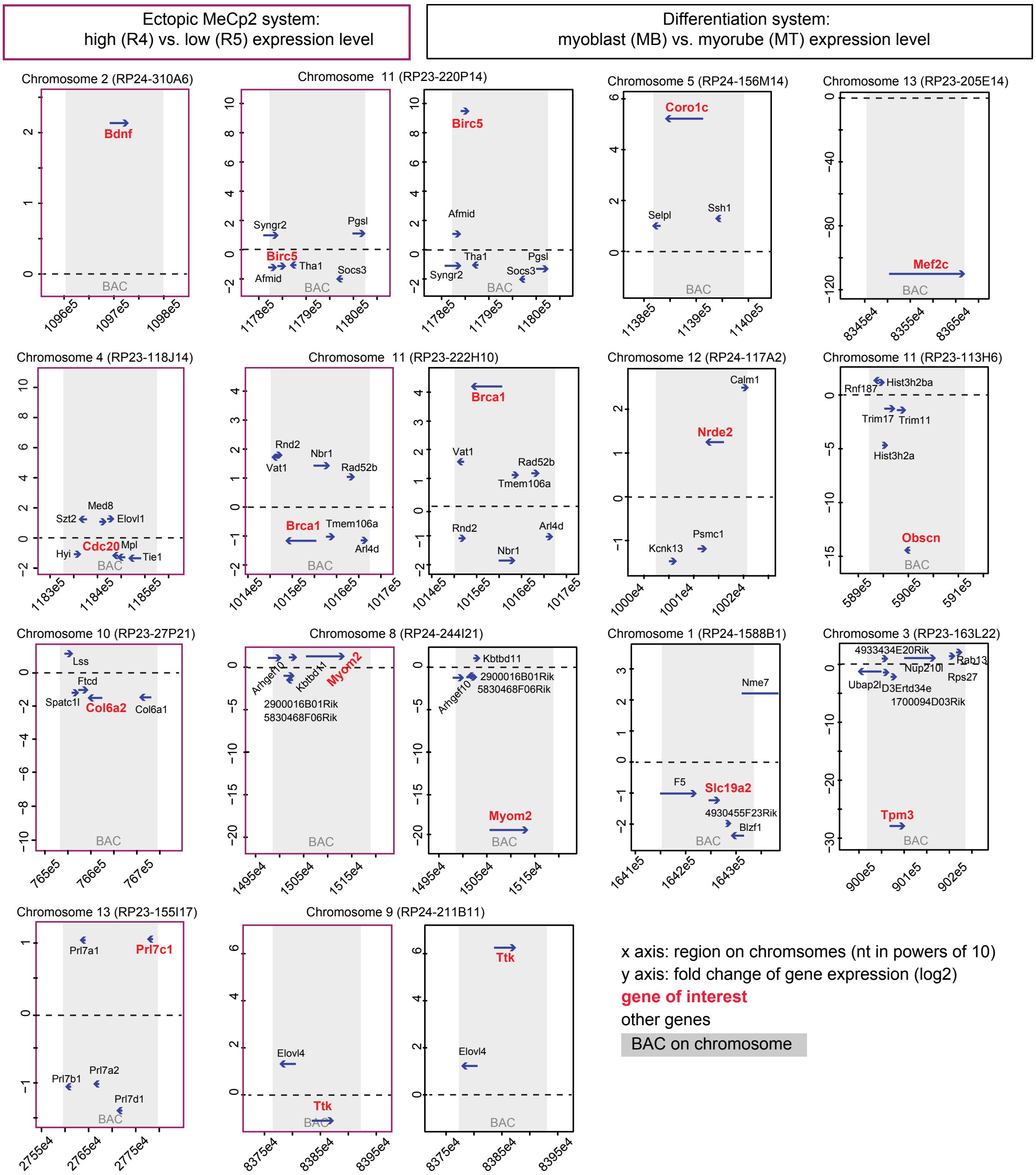


BAC IDs and mouse chromosome numbers are shown for each gene selected. The Y-axis indicates the expression fold change (-log 10) for each system (i.e., ectopic system: high level versus low level MeCP2; differentiation system: myotubes versus myoblasts). The X-axis gives the respective genomic location in powers of 10. Shaded boxes highlight the respective BAC region. Within BAC regions, genes are drawn to scale as blue arrows (orientation indicated by arrowheads) and the genes of interest are highlighted in red. Data sets boxed in pink highlight genes selected in the ectopic MeCP2 system; black boxes highlight data sets selected for the differentiation system. Genes selected in both systems are grouped in the middle with their individual expression fold changes for each system.

**Table S5. Genes analyzed during differentiation and their genomic context.**

| | Gene | *Mef2c* | *Tpm3* | ***Myom2*** | *Obscn* | *Slc19a2* | *Nrde2* | ***Brca1*** | *Coro1c* | ***Ttk*** | ***Birc5*** |
|---|---|---|---|---|---|---|---|---|---|---|---|
| | Maximum gene expression value | 109.95 | 27.91 | 19.12 | 14.44 | 1.22 | -1.25 | -4.13 | -5.44 | -6.26 | -9.48 |
| 2-Mbp window | Gene activity | 2.35 | 1.05 | 2.87 | 1.29 | -1.13 | -1.17 | 1.11 | 1,00 | -1.25 | -1.14 |
| | CpG island | 10 | 80 | 18 | 66 | 23 | 30 | 67 | 38 | 20 | 54 |
| | %GC content | 37.81 | 45.85 | 44.08 | 46.64 | 41.60 | 45.19 | 48.06 | 50.55 | 40.10 | 51.18 |
| | Gene density | 9 | 39 | 7 | 63 | 14 | 13 | 50 | 25 | 7 | 23 |
| | SINE | 3.22 | 15.88 | 3.54 | 11.45 | 6.35 | 10.27 | 21.70 | 12.04 | 5.19 | 11.87 |
| | LINE | 37.77 | 15.23 | 13.31 | 8.62 | 15.04 | 10.47 | 2.45 | 2.09 | 21.10 | 2.06 |
| 5-Mbp window | Gene activity | -1.01 | 1 | 1.34 | 1.11 | -1.06 | 1.06 | 1.01 | 1.05 | -1.17 | -1.01 |
| | CpG island | 24 | 147 | 58 | 117 | 63 | 63 | 127 | 99 | 35 | 162 |
| | %GC content | 38.34 | 44.13 | 44.15 | 46.35 | 41.84 | 43.72 | 47.08 | 49.80 | 39.05 | 50.7 |
| | Gene density | 32 | 186 | 47 | 157 | 82 | 60 | 221 | 105 | 29 | 153 |
| | SINE | 3.38 | 13.38 | 4.97 | 12.46 | 7.14 | 8.86 | 17.57 | 12.27 | 4.45 | 14.78 |
| | LINE | 31.27 | 19.02 | 11.16 | 8.95 | 15.86 | 16.85 | 4.82 | 3.21 | 25.54 | 1.88 |

**Table S6. Genes analyzed in MeCP2 expressing cells and their genomic context.**

| | Gene | *Bdnf* | *Prl7c1* | ***Myom2*** | *Cdc20* | ***Brca1*** | ***Birc5*** | ***Ttk*** | *Col6a2* |
|---|---|---|---|---|---|---|---|---|---|
| | Maximum gene expression value | 2.13 | -1.16 | 1.32 | -1.16 | -1.14 | -1.13 | -1.13 | -1.52 |
| 2-Mbp window | Gene activity | 1.51 | 1.34 | 1.05 | 1.06 | 1.04 | -1.07 | -1.42 | -1.13 |
| | CpG island | 12 | 25 | 18 | 49 | 67 | 54 | 20 | 36 |
| | %GC content | 38.98 | 42.02 | 44.08 | 47.15 | 48.06 | 51.18 | 40.10 | 47.42 |
| | Gene density | 9 | 24 | 7 | 34 | 50 | 23 | 7 | 21 |
| | SINE | 5.13 | 9.02 | 3.54 | 14.82 | 21.70 | 11.87 | 5.19 | 9.93 |
| | LINE | 25.65 | 21.75 | 13.31 | 8.07 | 2.45 | 2.06 | 21.10 | 8.53 |
| 5-Mbp window | Gene activity | 1.51 | 1.28 | -1.05 | 1.12 | 1.04 | -1.01 | 1,00 | -1.01 |
| | CpG islands | 27 | 38 | 58 | 128 | 127 | 162 | 35 | 76 |
| | %GC content | 38.09 | 39.16 | 44.15 | 46.14 | 47.08 | 50.7 | 39.05 | 45.73 |
| | Gene density | 77 | 57 | 47 | 143 | 221 | 153 | 29 | 131 |
| | SINE | 3.52 | 5.12 | 4.97 | 16.78 | 17.57 | 14.78 | 4.45 | 8.62 |
| | LINE | 32.35 | 28.27 | 11.16 | 7.40 | 4.82 | 1.88 | 25.54 | 14.70 |

The gene activity of genomic regions was calculated as the average of all Affymetrix probe sets overlapping with the corresponding genomic regions. The number of genes (gene density) and the number of CpG islands was retrieved from the Ref-genes and CpG entries respectively in the genome browser (m38 assembly) overlapping with the corresponding genomic coordinates. GC content (fraction of GC within sequence), LINE and SINE density (percentage of covered sequences) was calculated using the corresponding genomic regions submitted to RepeatMasker (http://www.repeatmasker.org, version open-4.0).

**Table S7. Chi square result for gene distributions[1].**

| | Chromocenter | | | | Periphery | | | |
|---|---|---|---|---|---|---|---|---|
| Gene | MB | MT | Low MeCP2 | High MeCP2 | MB | MT | Low MeCP2 | High MeCP2 |
| *Mef2c* | $2.4x10^{-05}$ | $3.9x10^{-04}$ | n/a | n/a | $6.7x10^{-09}$ | $7.2x10^{-13}$ | n/a | n/a |
| *Tpm3* | $1.3x10^{-07}$ | $1.3x10^{-03}$ | n/a | n/a | $9.4x10^{-09}$ | $4.4x10^{-12}$ | n/a | n/a |
| *Myom2* | $2.2x10^{-06}$ | $6.2x10^{-01}$ | $3.5x10^{-02}$ | $1.2x10^{-03}$ | $1.1x10^{-01}$ | $2.4x10^{-14}$ | $2.6x10^{-05}$ | $3.3x10^{-03}$ |
| *Obscn* | $3.8x10^{-01}$ | $5.2x10^{-04}$ | n/a | n/a | $5.0x10^{-08}$ | $2.6x10^{-13}$ | n/a | n/a |
| *Slc19a2* | $4.2x10^{-13}$ | $1.6x10^{-06}$ | n/a | n/a | $3.9x10^{-05}$ | $5.1x10^{-18}$ | n/a | n/a |
| *Nrde2* | $1.3x10^{-12}$ | $8.4x10^{-04}$ | n/a | n/a | $1.5x10^{-13}$ | $1.8x10^{-16}$ | n/a | n/a |
| *Brca1* | $8.0x10^{-09}$ | $2.8x10^{-05}$ | $6.8x10^{-03}$ | $1.7x10^{-03}$ | $4.3x10^{-14}$ | $3.7x10^{-31}$ | $1.1x10^{-28}$ | $3.0x10^{-22}$ |
| *Coro1c* | $1.6x10^{-04}$ | $3.8x10^{-07}$ | n/a | n/a | $6.1x10^{-10}$ | $2.7x10^{-06}$ | n/a | n/a |
| *Ttk* | $7.8x10^{-07}$ | $2.6x10^{-02}$ | $3.7x10^{-02}$ | $2.0x10^{-03}$ | $1.7x10^{-01}$ | $4.2x10^{-08}$ | $1.4x10^{-02}$ | $5.1x10^{-04}$ |
| *Birc5* | $6.5x10^{-05}$ | $1.5x10^{-08}$ | $1.0x10^{-03}$ | $4.4x10^{-07}$ | $8.4x10^{-18}$ | $7.6x10^{-17}$ | $5.4x10^{-12}$ | $6.1x10^{-22}$ |
| *Bdnf* | n/a | n/a | $4.5x10^{-09}$ | $8.8x10^{-11}$ | n/a | n/a | $7.0x10^{-02}$ | $6.3x10^{-05}$ |
| *Prl7c1* | n/a | n/a | $6.0x10^{-03}$ | $1.1x10^{-04}$ | n/a | n/a | $1.0x10^{-08}$ | $2.5x10^{-10}$ |
| *Cdc20* | n/a | n/a | $4.0x10^{-10}$ | $1.7x10^{-11}$ | n/a | n/a | $6.4x10^{-01}$ | $1.1x10^{-07}$ |
| *Col6a2* | n/a | n/a | $5.5x10^{-03}$ | $4.0x10^{-05}$ | n/a | n/a | $4.2x10^{-08}$ | $5.3x10^{-16}$ |

[1] Grey boxes identify random gene distribution.

**Figure S8. Exemplary analysis of neighborhood gene activity.**

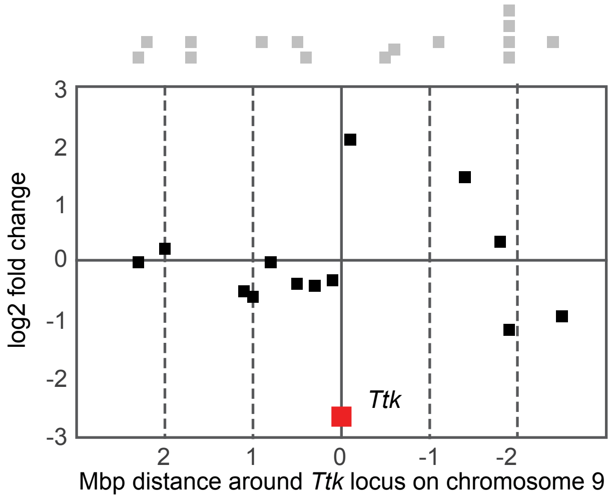


The average gene activity of genes in the neighborhood was calculated using the gene expression profiling data. The expression data however do not cover all genes in the region. Therefore, the gene activity may not necessarily reflect the gene activity of the complete neighborhood. An example on how such an activity profile looks is given. From the 29 genes in the 5-Mbp neighborhood (the x-axis shows 2.5 Mbp up and downstream from the gene center) of the *Ttk* protein kinase locus (red square), 15 were not identified by our myogenesis expression profile (grey squares) and 13 were identified (black squares). In the distribution of gene activities below one can see that an equaly strong upregulated gene lies right next to the gene of interest which could directly influence positional changes of *Ttk*.

**Table S8. Transcription profiling parameters and output.**

| File*** | Date | Chip | Alias | Outlier* | RawQ | SF | TGT | NF | Bckgrnd | Noise | No_P |
|---|---|---|---|---|---|---|---|---|---|---|---|
| CAR_01_MB1_190805_430_2.CHP | 11:44AM 08/23/2005 | Mouse430_2 | MB1 | 36 | 2.19 | 0.775 | 200 | 1 | 61.9 | 4.6 | 47.00% |
| CAR_02_MB2_190805_430_2.CHP | 11:44AM 08/23/2005 | Mouse430_2 | MB2 | 54 | 2.35 | 0.805 | 200 | 1 | 70.31 | 4.39 | 46.30% |
| CAR_03_MB3_190805_430_2.CHP | 11:44AM 08/23/2005 | Mouse430_2 | MB3 | 44 | 2.22 | 0.907 | 200 | 1 | 67.71 | 4.2 | 45.50% |
| CAR_04_MB4_190805_430_2.CHP | 11:44AM 08/23/2005 | Mouse430_2 | MB4 | 39 | 2.31 | 0.852 | 200 | 1 | 69.13 | 4.13 | 46.40% |
| CAR_05_MB6_190805_430_2.CHP | 11:44AM 08/23/2005 | Mouse430_2 | MB6 | 53 | 2.31 | 0.752 | 200 | 1 | 69.29 | 4.58 | 46.70% |
| CAR_06_MT1_190805_430_2.CHP | 11:44AM 08/23/2005 | Mouse430_2 | MT1 | 56 | 2.34 | 0.884 | 200 | 1 | 68.61 | 4.35 | 48.00% |
| CAR_07_MT_190805_430_2.CHP | 11:44AM 08/23/2005 | Mouse430_2 | MT0 | 34 | 2.3 | 0.775 | 200 | 1 | 69.04 | 4.68 | 47.90% |
| CAR_08_MT3_190805_430_2.CHP | 11:44AM 08/23/2005 | Mouse430_2 | MT3 | 52 | 2.1 | 0.94 | 200 | 1 | 65.56 | 4.02 | 46.80% |
| CAR_09_MT4_190805_430_2.CHP | 11:44AM 08/23/2005 | Mouse430_2 | MT4 | 40 | 2.34 | 0.72 | 200 | 1 | 65.25 | 4.59 | 49.30% |
| CAR_10_MT2_190805_430_2.CHP | 11:44AM 08/23/2005 | Mouse430_2 | MT2 | 109 | 2.31 | 0.881 | 200 | 1 | 66.71 | 4.95 | 46.90% |
| CAR_11_R5_1_240805_430_2.CHP | 01:28PM 08/25/2005 | Mouse430_2 | R5_1 | 29** | 3.85 | 0.802 | 200 | 1 | 128.29 | 12 | 39.40% |
| CAR_12_R5_3_240805_430_2.CHP | 01:30PM 08/25/2005 | Mouse430_2 | R5_3 | 104** | 4.36 | 0.903 | 200 | 1 | 157.98 | 13.15 | 37.90% |
| CAR_13_R5_5_240805_430_2.CHP | 01:30PM 08/25/2005 | Mouse430_2 | R5_5 | 32 | 2.46 | 0.891 | 200 | 1 | 74.9 | 4.72 | 42.90% |
| CAR_14_R5_1807_240805_430_2.CHP | 01:31PM 08/25/2005 | Mouse430_2 | R5_1807 | 15 | 2.67 | 1.065 | 200 | 1 | 85.87 | 5.87 | 41.80% |
| CAR_15_R5_10_240805_430_2.CHP | 01:32PM 08/25/2005 | Mouse430_2 | R5_10 | 32 | 2.34 | 0.994 | 200 | 1 | 73.42 | 4.78 | 42.70% |
| CAR_16_R4p1_240805_430_2.CHP | 01:33PM 08/25/2005 | Mouse430_2 | R4_1 | 35 | 2.41 | 1.01 | 200 | 1 | 74.51 | 4.83 | 42.70% |
| CAR_17_R4p2_240805_430_2.CHP | 01:34PM 08/25/2005 | Mouse430_2 | R4_2 | 114 | 2.24 | 0.936 | 200 | 1 | 69.63 | 4.55 | 45.30% |
| CAR_18_R4p3_240805_430_2.CHP | 01:36PM 08/25/2005 | Mouse430_2 | R4_3 | 35 | 2.24 | 1.039 | 200 | 1 | 69.85 | 4.28 | 42.60% |
| CAR_19_R4p4_240805_430_2.CHP | 01:36PM 08/25/2005 | Mouse430_2 | R4_4 | 53 | 2.28 | 1.052 | 200 | 1 | 70.69 | 4.1 | 42.70% |
| CAR_20_R4p1807_240805_430_2.CHP | 01:38PM 08/25/2005 | Mouse430_2 | R4_1807 | 46 | 2.38 | 1.1 | 200 | 1 | 73.25 | 4.73 | 43.40% |
| Minimum | | | | 15 | 2.1 | 0.72 | | | 61.9 | | 0.379 |
| Maximum | | | | 114 | 4.36 | 1.1 | | | 157.98 | | 0.493 |

*Number of outlier in the Nalimov test at $p < 0.001$

**No arrays have been rejected from the statistical analysis in respect to the fact that the quality of the sub-optimal arrays (red marked) is not clearly

| File | No_A | No_M | Avg_P | Avg_A | Avg_M | Avg_All | GAPDH | 18S | β_ACTIN | BIOB | BIOC |
|---|---|---|---|---|---|---|---|---|---|---|---|
| CAR_01_MB1_190805_430_2.CHP | 51.30% | 1.60% | 590.5 | 28 | 76.2 | 293.4 | 0.86 | 0.32 | 1.16 | 0.83 | 1.11 |
| CAR_02_MB2_190805_430_2.CHP | 51.90% | 1.70% | 619.3 | 25.9 | 73.4 | 301.7 | 0.84 | 0.28 | 1.16 | 0.71 | 1.03 |
| CAR_03_MB3_190805_430_2.CHP | 53.00% | 1.60% | 636.3 | 27.7 | 82.7 | 305.4 | 0.82 | 0.33 | 1.15 | 0.92 | 1.1 |
| CAR_04_MB4_190805_430_2.CHP | 51.90% | 1.70% | 618.9 | 26.8 | 77.4 | 302.3 | 0.81 | 0.38 | 1.14 | 0.75 | 1.05 |
| CAR_05_MB6_190805_430_2.CHP | 51.70% | 1.60% | 604.1 | 24.5 | 67.5 | 295.8 | 0.82 | 0.42 | 1.13 | 0.76 | 1.08 |
| CAR_06_MT1_190805_430_2.CHP | 50.30% | 1.70% | 574.6 | 28.5 | 73.5 | 291.1 | 0.81 | 0.53 | 1.17 | 0.8 | 0.96 |
| CAR_07_MT_190805_430_2.CHP | 50.30% | 1.70% | 577.1 | 25.6 | 68.7 | 290.8 | 0.81 | 0.58 | 1.17 | 0.78 | 1.01 |
| CAR_08_MT3_190805_430_2.CHP | 51.60% | 1.60% | 604.1 | 26.5 | 70.7 | 297.6 | 0.8 | 0.52 | 1.19 | 0.76 | 1.03 |
| CAR_09_MT4_190805_430_2.CHP | 48.90% | 1.80% | 537.8 | 28.8 | 89.9 | 280.7 | 0.79 | 0.62 | 1.13 | 1.18 | 1.02 |
| CAR_10_MT2_190805_430_2.CHP | 51.30% | 1.70% | 579.9 | 30.3 | 83.1 | 289.1 | 0.79 | 0.62 | 1.26 | 0.79 | 1.07 |
| CAR_11_R5_1_240805_430_2.CHP | 58.90% | 1.70% | 676.5 | 38.3 | 121.7 | 291.3 | 1.82 | 0.35 | 1.61 | 0.81 | 0.75 |
| CAR_12_R5_3_240805_430_2.CHP | 60.40% | 1.70% | 696.8 | 44.9 | 130.4 | 293.7 | 1.95 | 0.27 | 1.91 | 0.69 | 0.8 |
| CAR_13_R5_5_240805_430_2.CHP | 55.50% | 1.70% | 678.6 | 26.6 | 82.4 | 307.2 | 1.92 | 0.3 | 1.61 | 0.93 | 0.87 |
| CAR_14_R5_1807_240805_430_2.CHP | 56.60% | 1.60% | 694.5 | 31.2 | 87.8 | 309.4 | 2.06 | 0.23 | 1.66 | 0.94 | 0.88 |
| CAR_15_R5_10_240805_430_2.CHP | 55.60% | 1.70% | 680.8 | 27.2 | 85.8 | 307.4 | 1.71 | 0.22 | 1.67 | 0.75 | 1.15 |
| CAR_16_R4p1_240805_430_2.CHP | 55.60% | 1.70% | 687.7 | 26.8 | 82.4 | 310 | 1.77 | 0.27 | 1.83 | 0.81 | 1.25 |
| CAR_17_R4p2_240805_430_2.CHP | 53.10% | 1.60% | 661.1 | 22.1 | 62.7 | 312.3 | 1.93 | 0.3 | 1.9 | 0.96 | 1.16 |
| CAR_18_R4p3_240805_430_2.CHP | 55.80% | 1.60% | 693.8 | 26.4 | 76.1 | 311.5 | 1.96 | 0.25 | 1.71 | 0.75 | 1.04 |
| CAR_19_R4p4_240805_430_2.CHP | 55.70% | 1.60% | 691.5 | 25.8 | 90.2 | 310.9 | 2.25 | 0.26 | 1.76 | 0.91 | 1.14 |
| CAR_20_R4p1807_240805_430_2.CHP | 55.00% | 1.70% | 687.8 | 25.1 | 73.9 | 313.2 | 2.15 | 0.25 | 1.6 | 1.07 | 1.16 |
| Minimum | | | 537.8 | | | | 0.79 | | 1.13 | | |
| Maximum | | | 696.8 | | | | 2.25 | | 1.91 | | |

| File | BIOD | CRE | DAP | LYS | PHE | THR | TRP | Alpha1 | Alpha2 | Tau |
|---|---|---|---|---|---|---|---|---|---|---|
| CAR_01_MB1_190805_430_2.CHP | 2.28 | 1.32 | 0.25 | 1.9 | 30.66 | 2.23 | 1.95 | 0.05 | 0.065 | 0.015 |
| CAR_02_MB2_190805_430_2.CHP | 2.05 | 1.31 | 0.6 | 0.5 | 11.01 | 0.2 | 0.67 | 0.05 | 0.065 | 0.015 |
| CAR_03_MB3_190805_430_2.CHP | 2.57 | 1.39 | 0.26 | 2 | 6.46 | 0.9 | 0.79 | 0.05 | 0.065 | 0.015 |
| CAR_04_MB4_190805_430_2.CHP | 2.4 | 1.3 | 2.31 | 1.04 | 10.33 | 1.58 | 0.09 | 0.05 | 0.065 | 0.015 |
| CAR_05_MB6_190805_430_2.CHP | 2.11 | 1.32 | 0.63 | 0.61 | 27.86 | 1.38 | 0.18 | 0.05 | 0.065 | 0.015 |
| CAR_06_MT1_190805_430_2.CHP | 2.29 | 1.23 | 0.35 | 2.46 | 33.86 | 0.49 | 1.64 | 0.05 | 0.065 | 0.015 |
| CAR_07_MT_190805_430_2.CHP | 1.95 | 1.16 | 1.06 | 0.3 | 19.17 | 0.56 | 0.49 | 0.05 | 0.065 | 0.015 |
| CAR_08_MT3_190805_430_2.CHP | 2.34 | 1.25 | 2.3 | 11.72 | 25.17 | 1.15 | 0.39 | 0.05 | 0.065 | 0.015 |
| CAR_09_MT4_190805_430_2.CHP | 1.89 | 1.28 | 0.13 | 0.84 | 18.21 | 0.78 | 0.56 | 0.05 | 0.065 | 0.015 |
| CAR_10_MT2_190805_430_2.CHP | 2.37 | 1.18 | 1.17 | 9.14 | 30.6 | 0.51 | 1.05 | 0.05 | 0.065 | 0.015 |
| CAR_11_R5_1_240805_430_2.CHP | 2.38 | 1.16 | 16.68 | 14 | 6.93 | 7.19 | 0.19 | 0.05 | 0.065 | 0.015 |
| CAR_12_R5_3_240805_430_2.CHP | 2.37 | 1.21 | 14.75 | 49.1 | 15.92 | 17.66 | 0.31 | 0.05 | 0.065 | 0.015 |
| CAR_13_R5_5_240805_430_2.CHP | 2.42 | 1.24 | 21.04 | 13.27 | 13.45 | 7.87 | 0.67 | 0.05 | 0.065 | 0.015 |
| CAR_14_R5_1807_240805_430_2.CHP | 2.78 | 1.29 | 24.49 | 9.26 | 9.92 | 6.76 | 1.55 | 0.05 | 0.065 | 0.015 |
| CAR_15_R5_10_240805_430_2.CHP | 2.87 | 1.18 | 19.31 | 12.92 | 7.44 | 5.85 | 0.1 | 0.05 | 0.065 | 0.015 |
| CAR_16_R4p1_240805_430_2.CHP | 2.82 | 1.21 | 22.2 | 10.09 | 6.73 | 18.36 | 0.79 | 0.05 | 0.065 | 0.015 |
| CAR_17_R4p2_240805_430_2.CHP | 2.63 | 1.15 | 18.8 | 6.34 | 8.56 | 3.89 | 0.06 | 0.05 | 0.065 | 0.015 |
| CAR_18_R4p3_240805_430_2.CHP | 2.75 | 1.21 | 25.14 | 14.2 | 6.87 | 10.17 | 0.07 | 0.05 | 0.065 | 0.015 |
| CAR_19_R4p4_240805_430_2.CHP | 2.57 | 1.25 | 32.03 | 30.61 | 6.81 | 7.43 | 0.12 | 0.05 | 0.065 | 0.015 |
| CAR_20_R4p1807_240805_430_2.CHP | 2.68 | 1.28 | 26.04 | 12.09 | 12.94 | 8.28 | 0.13 | 0.05 | 0.065 | 0.015 |
| Minimum | | | | | | | | | | |
| Maximum | | | | | | | | | | |
